# Diabetes Mellitus: Review of the Common, Rare, and Uncommon (Conceptual and Metaphorical) Types

**DOI:** 10.3390/ijms27219611

**Published:** 2026-10-27

**Authors:** Monika Pliszka, Leszek Szablewski

**Affiliations:** Department of General Biology and Parasitology, Medical University of Warsaw, Chałubińskiego Str. 5, 02-004 Warsaw, Poland

**Keywords:** diabetes mellitus characteristics, types of diabetes mellitus, common types, rare types, not formally diagnosed

## Abstract

Diabetes mellitus (DM) comprises a complex heterogeneous group of chronic metabolic disorders that affect various organ systems, resulting in a number of complications, including kidney disease, cardiovascular disease, immune dysfunction, retinopathy, neuropathy, and cancer. Uncontrolled DM may also lead to a coma and confusion, and if untreated, death due to ketoacidosis or nonketotic hyperosmolar syndrome. In 2019, DM was directly responsible for 1.5 million deaths, with an additional 460,000 due to kidney disease, as well as about 20% of cardiovascular-associated deaths. In addition to the well-known types—*viz*. type 1 diabetes mellitus (T1DM), type 2 diabetes mellitus (T2DM), and gestational diabetes mellitus (GDM)—several other rare conceptual/metaphoric forms have been described, although they are not regarded as formal diagnoses. The aim of this review is to present the various types of DM and summarize their characteristics, clinical features, diagnosis, pathophysiology, risk factors, management, and therapy.

## 1. Introduction

The term diabetes mellitus (DM) comprises a group of metabolic disorders associated with dysfunctions in glucose synthesis by gluconeogenesis and glycogenolysis, resulting in the overproduction of glucose and its underuse as an energy source. The resulting persistent elevation in blood glucose level is known as hyperglycemia [1]. The effects of diabetes mellitus are also associated with the activity of insulin, an anabolic hormone that influences the metabolism of carbohydrates, lipids, and proteins [2]. A lack of insulin secretion, its impaired activity, or both may result in chronic hyperglycemia.

The metabolic disturbances associated with DM mainly affect tissues and organs, such as adipose tissue, skeletal muscles, and the liver, due to insulin resistance (IR), with the severity of symptoms depending on the duration and type of DM. For example, diabetic patients with a complete lack of insulin, as may be the case in children, may experience increased appetite, polydipsia, dysuria, weight loss, and vision problems. In contrast, those with early type 2 diabetes mellitus may not experience any symptoms [3]. However, if uncontrolled, DM may result in severe symptoms such as a coma and confusion. In rare cases, without proper treatment, DM can lead to death due to ketoacidosis or nonketotic hyperosmolar syndrome [2].

The global prevalence of DM is growing [4]. Based on World Health Organization (WHO) data, in 2014, 8.5% of adults aged 18 years or over were reported to be affected by DM. In 2019, DM was the cause of 1.5 million deaths, with 48% of these occurring before the age of 70 years; it was also responsible for 460,000 deaths caused by kidney disease and about 20% of cardiovascular-related deaths, associated with chronic hyperglycemia. In addition, a 3% rise in standardized mortality rates associated with DM was reported over the ten-year period from 2010 to 2019 [5].

Currently, DM is classified as T1DM, T2DM, GDM, monogenic diabetes, exocrine pancreatic disorders, and high-risk medications; however, this classification is being reconsidered following new data on inter alia physiology, genetics, and metabolomics [3,6,7,8].

A diagnosis of DM may be based on HbA1c or plasma glucose criteria. Plasma glucose criteria include fasting plasma glucose (FPG) level and two-hour plasma glucose (2 h PG) level during a 75 g oral glucose tolerance test (OGTT); it can also be indicated by characteristic symptoms of hyperglycemia, such as polyuria, polydipsia, unexplained weight loss, or a hyperglycemic crisis, such as diabetic ketoacidosis (DKA) or a hyperglycemic hyperosmolar state (HHS) [6].

The aim of this review is to present the different types of DM and their clinical significance. Diabetes mellitus is classified as common, rare, and uncommon (i.e., conceptual and metaphorical) types based on its frequency. The paper also summarizes the prevalence, diagnosis, pathophysiology, complications, and risk factors associated with each type and describes its management and therapy; in some cases, this description is accompanied by the characteristic symptoms that can be used to differentiate one type from the others.

## 2. Characteristics of Types of DM

Diabetes mellitus is classified conventionally into several categories. The two most common forms are T1DM and T2DM, with the latter being more prevalent, while GDM is associated with pregnancy. However, other forms of DM have been recorded, including latent autoimmune diabetes in adults (LADA), maturity-onset diabetes of the young (MODY), pancreatogenic DM (T3cDM), and secondary to various medications, such as corticosteroids [3,4,8]. These types of DM differ in their prevalence, diagnosis, pathogenesis, and therapy (Table 1).

### 2.1. Common Types of Diabetes Mellitus

#### 2.1.1. Type 2 Diabetes Mellitus

Type 2 diabetes mellitus (T2DM), also known as *non-insulin-dependent diabetes mellitus* (NIDDM) or *adult-onset diabetes mellitus*, is the most common type of DM, accounting for about 90% of all cases of DM. While this type is most commonly diagnosed in patients over 45 years of age, its prevalence among children, adolescents, and younger adults is increasing. This increase is associated with the growing prevalence of obesity, sedentary lifestyles, physical inactivity, and high-energy diets. It is characterized by persistent elevated blood glucose (hyperglycemia) or increased blood glucose levels after a meal containing carbohydrates [9]. Most patients with T2DM exhibit elevated blood insulin levels (hyperinsulinemia) during fasting and/or post-glucose ingestion [10]. This has been attributed to *insulin resistance* (IR), a term coined to account for the persistent elevation of glucose levels observed despite the presence of insulin [11]. Patients with T2DM also demonstrate pancreatic β-cell dysfunction [12,13].

##### Prevalence of T2DM

According to the NCD Risk Factor Collaboration 2022 data, approximately 828 million individuals worldwide are diagnosed with DM, of which over 95% are classified as T2DM [14]. Worryingly, the number of cases of T2DM is predicted to rise from around 366 million people aged 20 to 79 years (8.3%) in 2011 to around 439 million by the year 2030 [15]. Other estimates predict a rise in total T2DM cases from 2.8% of the global population (181 million) in 2000 to 4.4% (366 million) in 2030 [16] and the number of patients with T2DM to 700 million by 2045 [17]. In developing countries, the majority of diabetic patients are aged between 45 and 64 years [16]. The greatest number of patients with T2DM are aged 40–59 years.

The incidence and prevalence of T2DM depend on the geographical region, with more than 80% of cases being diagnosed in low-to-middle-income countries. The highest prevalence has been reported in the Eastern Mediterranean and the Middle East (9.2% of adult population), as well as in North America (8.4%). The highest number of diagnosed patients with T2DM is found in the Western Pacific (67 million diabetic patients) and Europe (53 million). In India, the highest number of diabetic patients was detected (40.9 million), following by China (39.8 million), the USA, Russia, Germany, Japan, Pakistan, Brazil, Mexico, and Egypt. Developing countries account for seven of the world’s top diagnoses. The greatest increase in DM incidences is observed in the Middle East, India, China, and the USA [18]. More recent observations confirm that nearly half of all adult patients with T2DM live in India and China [19].

##### Diagnosis of T2DM

A formal diagnosis of T2DM is based on American Diabetes Association (ADA) or WHO National Diabetes Group criteria of 2006 [20]. The classic symptoms include polyuria, polydipsia, polyphagia, growth impairment, blurred vision, and weight loss. These can be supported by the HbA1c criteria or glucose concentration measurements, *viz*. FPG, OGTT, and random plasma glucose concentration [20,21,22].

The HbA1c test is a simple procedure that can be used to monitor glycemic levels. It has been found to be effective in identifying patients with DM or those at risk of complications due to DM [23]. The FPG test is used to determine blood glucose levels. It is administered in the morning after an eight-hour overnight fast. According to ADA recommendations, FPG level ≥ 126 mg/dL (≥7.0 mmol/L) suggests DM [24]. The oral glucose challenge (OGTT) is used to screen for T2DM. The test is performed by measuring the plasma glucose level before administration of 75 g of glucose and comparing it with the level two hours later. The result indicates how fast glucose can be cleared from the blood, with a plasma glucose level of 200 mg/dL or above in the second sample indicating DM. Finally, in the random plasma glucose test, the blood glucose level is measured after eating a meal; a blood glucose level ≥ 200 mg/dL indicates DM, particularly when accompanied by classic symptoms of hyperglycemia crisis [21], such as increased thirst, hunger, and urination. While most patients with T2DM tend to be older, the incidence of T2DM among people under 40 years of age has been increasing. However, among younger patients, it is important to exclude other types of DM, such as T1DM, LADA, and MODY, before a correct diagnosis of T2DM can be made [21].

##### Pathophysiology of T2DM

Cases where T2DM is more developed are characterized by IR in peripheral tissues and impaired insulin production [19,25]. Insulin resistance is defined as the loss of the ability of target tissues, such as skeletal, adipose, and hepatic tissues, to respond to an insulin signal. Reduced insulin action causes hyperinsulinemia, leading to abnormalities in glucose, lipid, and protein metabolism [26], as well as obesity, metabolic dysfunction-associated steatotic liver diseases (MASLD), and hypertension [27].

The occurrence of IR depends on sex and ethnicity. For example, women tend to exhibit a lower prevalence of T2DM and IR, associated with disease at a younger age, and decreased mortality due to DM [28]. Also, IR is approximately two to three times more common in Asian Indians than in other races [29]. The action of insulin is regulated by various hormones, such as insulin-like growth factor 1 (IGF-1) in the fed state and glucagon, glucocorticoids, and catecholamines in the fasting state; any disturbances in their production may influence the development of IR [17,30]. Hormonal changes throughout puberty are also believed to increase the risk of IR. For example, an increase in the level of growth hormone (GH) followed by extensive lipid breakdown increases free fatty acid (FFA) concentrations in the bloodstream; this is regarded as a key factor in short-term increases in IR [31].

Generally, IR is most commonly observed in the skeletal muscle, liver, and adipose tissue; among these, IR in the skeletal muscle plays an important role in the development of T2DM. In the physiological state, insulin stimulates the synthesis of glycogen from glucose and its uptake from the bloodstream [32]. During this process, glucose transporter 4 (GLUT4) is translocated from intracellular compartments into the cell membrane via the intracellular insulin signaling pathway. This pathway is impaired by IR, resulting in decreased GLUT4 expression and translocation and causing hyperglycemia due to reduced cellular glucose uptake. Impaired GLUT4 function also decreases glycogenesis, which is regarded as the primary metabolic defect in the pathogenesis of T2DM [33]. In adipose tissue, IR suppresses lipolysis, decreases glucose uptake, and increases the release of FFAs into the blood. The presence of elevated levels of circulating FFAs promotes ectopic fat deposition around the skeletal muscle and liver and impairs the insulin signaling pathway. In the liver, IR impairs glycogen and glucose metabolism while stimulating lipogenesis and the synthesis of pro-inflammatory cytokines [34]; the resulting systemic inflammation can exacerbate the impaired insulin response induced by the liver [34]. In the liver, IR increases the production of glucose after a night of fasting and reduces the suppression of glucose after a meal; the increased hepatic production of glucose can be associated with enhanced gluconeogenesis [35]. Finally, increased levels of FFAs in plasma may influence gluconeogenesis by activating certain gluconeogenic enzymes. For example, increased hepatic glucose-6-phosphatase activity has been noted in diabetic patients, resulting in increased glucose production [36]; this information is presented in more detail elsewhere [21,26,27,37,38,39,40].

Pancreatic β-cell dysfunction has also been associated with a reduction in insulin secretion, relative to insulin sensitivity. Patients with T2DM can also present defective and delayed secretion of insulin and abnormal postprandial suppression of glucagon, which may contribute to postprandial hyperglycemia.

The insulin response to a glucose challenge is biphasic. Initially, approximately two to four minutes after the initial rise of blood glucose levels, an acute insulin secretion phase can be observed. This is followed by a sharp drop in secretion lasting approximately 10 to 15 min. The second insulin secretion phase, i.e., the late phase, is more gradual and persists for as long as hyperglycemia is present; it reaches a steady state at two to three hours after the initial rise in glucose levels [41].

In patients with IR, pancreatic islet β-cells are stimulated to secrete higher levels of insulin [41]. The combination of insufficient insulin secretion and accompanying IR, glucotoxicity, and obesity-related inflammation can result in the development of hyperglycemia and, eventually, T2DM [42].

In patients with T2DM, the first phase is markedly reduced or sometimes even non-existent [43], and the second phase may often be delayed. Furthermore, most diabetic patients fail to demonstrate physiological oscillatory secretion with a periodicity of 12 to 15 min [44,45]. Hence, it has been proposed that reduced insulin pulsatility may be associated with impaired β-cell function [46].

Islet β-cell dysfunction is associated with impaired insulin synthesis, insulin secretion, and reduced β-cell mass and is believed to contribute to the progression of T2DM. Indeed, the pancreatic β-cell mass in patients with T2DM has been found to be 50% lower than that of healthy individuals [47]. This loss of β-cell mass has been attributed to β-cell exhaustion due to remaining in a state of prolonged elevated glucose metabolism and insulin secretion; as a result, the β-cells undergo apoptosis as an effect of glucotoxicity and lipotoxicity [48,49].

To compensate for increased IR, pancreatic β-cells increase their secretion of insulin. However, over time, the secretory response is impaired as a result of glucotoxicity, endoplasmic reticulum stress, mitochondrial dysfunction, and inflammation. Indeed, β-cell function impairment has been found to differ between later-onset and early-onset T2DM: studies on younger and adolescent patients with T2DM identified 20–35% deterioration of β-cell function in early-onset T2DM compared to 7% per year in later-onset T2DM [50]. The mechanisms behind the loss of β-cell function in early-onset T2DM are not entirely understood [37].

It has been hypothesized that compared to older adults, children and adolescents at the initial stages of impaired glucose tolerance (IGT) or newly-diagnosed T2DM exhibit more pronounced insulin secretion and faster exhaustion of β-cells [51], which express GH, estrogen, and androgen receptors; this increased exhaustion may be caused by the increased levels of hormones associated with growth and puberty [31]. Indeed, healthy children also exhibit a 30–50% fall in insulin sensitivity during puberty; it is possible that this hormonal stimulation may serve as a compensatory mechanism in individuals prone to β-cell dysfunction [52]. During T2DM, however, this compensatory increase in secretion may change to a reduction, together with a decrease in β-cell mass [53]. In the early stage of T2DM, β-cell dysfunction is associated with impaired synthesis and secretion of insulin. As mentioned above, β-cell survival is strongly influenced by chronic exposure to high glucose and lipids.

Hyperglycemia is associated with the continuous secretion of insulin. However, continual exposure to high glucose levels by β-cells results in reduced expression of insulin synthesis transcription factors, such as PDX1 and MAFA, and thus lower insulin synthesis. In diabetic patients, GLUT1 and GLUT2 expression is decreased, causing reduced transport of glucose into cells, decreased ATP production, and impaired insulin release.

Excess levels of non-esterified fatty acids (NEFAs) cause lipotoxicity. This stimulates insulin secretion and disrupts β-cell function by increasing basal insulin levels but inhibits glucose-stimulated insulin secretion (GSIS) [54]. Furthermore, the presence of saturated fatty acids (SFAs) increases the production of ceramide and NO, which disrupts insulin synthesis and induces β-cell apoptosis by inflammation, endoplasmic reticulum stress, and oxidative stress [54]. These factors are believed to be primary causes of β-cell dysfunction by apoptosis, although recent findings suggest that the cells are also subject to other forms of death, such as dedifferentiation, senescence, autophagy, pyroptosis, and ferroptosis [27,55,56]. This is covered in more detail in previous studies [17,21,27,37,57].

##### Complications of T2DM

According to data from the Emerging Risk Factors Collaboration, patients with T2DM might die up to six years earlier than those without [58]. Similarly, the Swedish National Diabetes Registry reports that patients diagnosed with young-onset T2DM at 15 years of age might die up to 12 years earlier than those without T2DM [59]. This increased risk of mortality is associated with an elevated risk of T2DM complications in comparison with usual-onset T2DM [19,59,60,61,62].

- Macrovascular complications

Diabetic patients are more susceptible to restenosis following vascular surgeries, such as angioplasties or stenting [63], with an approximately 43% greater risk of developing primary restenosis following endovascular therapy [64]. Also, the pathological features associated with DM, such as inflammation, endothelial cell dysfunction, lipid metabolism disturbances, and high blood pressure, also place the patient at a higher risk of cerebrovascular disease, such as ischemic stroke [64]. Diabetic patients are also at a higher risk of macrovascular complications such as peripheral artery disease. This disease is mainly diagnosed in the lower part of body, e.g., in the tibial vessels, which might manifest as medial calcification of the arterial walls, also known as Mönckeberg’s disease sclerosis [65]. A 1% increase in HbA1c is associated with as much as a 30% increase in the risk of PAD [66]. Also, T2DM is associated with two- to fourfold greater the mortality rate of adults caused by heart disease and stroke [67,68].

- Microvascular complications

Approximately 40% of patients with T2DM also present with diabetic kidney disease (DKD) [69], the most common form of chronic kidney disease (CKD), which is responsible for 50% of end-stage renal disease (ESRD) cases worldwide. Diabetic patients with DKD are at a 13% higher risk of death caused by cardiovascular disease (CVD) compared to diabetic patients without DKD [70]. This has been attributed to injury to the glomerular microvasculature and the development of diabetic nephropathy [71].

- Neuropathy

Many patients with T2DM also experience neuropathy [72], i.e., damage to the metabolic nerve and the tiny vessels of the nerves [73]. The condition is associated with thickening of the subcutaneous membrane, impaired blood circulation, and deficits in the capillaries, which nourish the nerves; this results in hypoxia of the tissue and peripheral nerve depletion, leading to motor, sensory, and autonomic disorders [64]. Neuropathy itself manifests as pain, tingling, paresthesia, numbness, and increased sensitivity to stimuli [74].

Almost 60% of patients with T2DM experience diabetic retinopathy (DR) after 20 years of disease duration [75]. The incidence of DR differs between disease types, being three times more common in T1DM than T2DM. It is also more common in women, but its course is worse in men [76]. The symptoms associated with DR include blurred vision, distorted vision, and partial or complete loss of vision [77]. This is described in more detail elsewhere [17,21,64].

##### Risk Factors for T2DM

One of the key pathological factors associated with the development of T2DM is the Western diet, i.e., a high-calorie diet that considerably increases blood sugar levels [52]. This diet contains chylomicrons and their remnants: these form triglycerides (TGs), circulating very-low-density lipoproteins (VLDLs), and elevated amounts of carbohydrates and lipids. The diet is also associated with elevated blood sugar levels and cholesterol levels, particularly among individuals with overweight and obesity. Those consuming a Western diet are also subject to increased levels of reactive oxygen species (ROS), which promote inflammation. Indeed, chronic elevation of ROS levels is regarded as an important factor in the development T2DM and IR. The pro-oxidant environment associated with ROS generates superoxide (O2) stress within the endoplasmic reticulum and mitochondrial dysfunction. The diet is also responsible for elevated levels of blood fatty acids, which are known to play a role in mitochondrial dysfunction [64].

Obesity and T2DM are associated with elevated indicators of continuous low-grade chronic inflammation. Decreased exercise and physical activity and a sedentary, inactive lifestyle are associated with higher levels of pro-inflammatory interleukin-6 (IL-6), IL-1, and tumor necrosis factor-α (TNF-α) in the bloodstream, resulting in metabolic inflammation [64]. In addition, β-cell function may also be impaired by other factors, such as by *disallowed genes* [78], which are upregulated during states of metabolic stress, e.g., T2DM; this is accompanied by the downregulation of the proper markers of pancreatic β-cells [52]. Several disallowed genes have been identified: one is believed to encode the repressor element 1 silencing transcription factor (*REST*) gene, whose repression is needed to allow the physiological secretion of insulin [78]; in addition, its overexpression decreases functional β-cell mass and causes T2DM [79]. When upregulated, the *REST* gene also activates other genes, which repress β-cell proliferation, thus impairing β-cell compensation in T2DM [78].

Other risk factors include aging and comorbid diseases [18,21,64].

##### Management and Therapy

In conventional therapy, the initial treatment goals concern improving diet and exercise and administering anti-hyperglycemic agents. While first-line pharmacological therapy typically consists of metformin [80], a range of other options are available. One such group of drugs includes sodium/glucose co-transporter 2 (SGLT2) inhibitors, which lower serum glucose levels by blocking the movement of glucose from the renal tubule into the epithelial lining [81,82]. Glucagon-like peptide 1 (GLP-1) agonists stimulate glucose-dependent secretion of insulin by pancreatic β-cells, promote β-cell neogenesis, and inhibit β-cell apoptosis. Dipeptidyl peptidase-4 (DPP-4) inhibitors block the action of DPP-4, which degrades incretins such as GLP-1; these drugs are also used in insulin therapy. Recent developments have added a number of other agents to the armory of potential T2DM treatments, such as dual incretin receptor agonists (GLP-1/GIP), GIP receptor triagonists, SGLT1/2 (dual inhibition), and glucagon receptor agonists. Alternatively, non-pharmacological options are available, such as exercise and dietary interventions, bariatric surgery, hyperbaric oxygen therapy, and probiotics [19,21,80].

#### 2.1.2. Type 1 Diabetes Mellitus

Type 1 diabetes mellitus (T1DM), previously known as *insulin-dependent diabetes* or *juvenile-onset diabetes* [7], is an autoimmune disease causing metabolic and nutritional complications associated with the destruction of insulin-producing pancreatic islet β-cells. Also, because most cases were diagnosed in childhood, the condition was also termed *juvenile diabetes*. Recent data indicate that nearly half of all T1DM cases are diagnosed in adulthood [83,84,85]. Worryingly, in at least one-third of cases, adult-onset T1DM is misclassified as T2DM, which delays appropriate treatment [85].

##### Prevalence of T1DM

It is estimated that T1DM accounts for about 5 to 10% of all diabetic patients [4]. It is the third most common chronic disease of childhood, affecting 1 in 300 children [86], and is typically diagnosed in children aged below seven years [86,87]. The lowest prevalence was reported in Canada in children aged under four years (<1:6000) [88]. The incidence of disease worldwide increased by 2.8% per year from 1990 to 1999 [89,90]; around 200 million people were estimated to have T1DM in 2000, and the number of cases is expected to double in 2030 [16]. In 2021, there were an estimated 572,220 cases of patients, including adolescents, in the world [91].

Globally, the annual incidence of T1DM varies from 5 to 42 per 100,000 individuals and the prevalence from 69 to 198.4, depending on region and population type. The highest incidences have been noted in Finnish, Sardinian, North Asian, and Northern European ethnic groups. The prevalence was also found to range from 0.6/1000 people in Asia, 0.8/1000 people in Africa, and 3.0/1000 people in Europe to 4.4/1000 people in North America. The incidence of T1DM is highest in higher income countries [86] and among men [84].

##### Diagnosis of T1DM

A diagnosis of T1DM is based on an FPG concentration >7.0 mmol/L (>126 mg/dL). Most individuals continue to be diagnosed based on the following clinical criteria proposed by the ADA: *viz*. random blood glucose concentration ≥11.1 mmol/L (≥200 mg/dL), accompanied by classic symptoms of hyperglycemia, such as polyuria, polydipsia, and unexplained weight loss; approximately a third of patients present with DKA [92]. In the absence of classic symptoms, a diagnosis may be established based on a HbA1c concentration ≥6.5% (≥48 mmol/mol) [6,86,93]. The progression of dysglycemia can be rapid in patients with T1DM, and HbA1c is less sensitive for diagnosis compared to fasting or stimulated blood glucose measurements [94]. In adults, the onset of T1DM can be variable, and the classic symptoms observed in children may not be present [93].

As both T1DM and T2DM share the same ADA diagnostic criteria [8], correct identification of diabetes type is difficult, and up to 40% of adult patients with T1DM are believed to be misclassified [95]. Misclassification has been attributed to various causes. Firstly, while T1DM accounts for over 90% of all DM cases in children, it only constitutes 5 to 10% of all cases of DM in adults [85]; secondly, the overwhelming majority of older adults have T2DM; thirdly, the clinical characteristics of adult-onset T1DM can masquerade as T2DM: for example, only 21% of adult patients with T1DM have HbA1c of less than 7% [86], and ketoacidosis observed in patients with T1DM may also occur in those who are ketosis-prone [96]; finally, clinical criteria such as BMI and the presence of metabolic syndrome are poor discriminators due to the high prevalence of obesity in the general population [95].

Hence, no single clinical feature can perfectly distinguish T1DM from T2DM at diagnosis. Rather, diagnosis depends on a combination of factors, such as the age of diagnosis, BMI, and the presence of biomarkers, such as pancreatic autoantibodies [97]. While no accepted specific criteria for the diagnosis of T1DM currently exist, two main features have been proposed for patients aged ≤20 years: *viz*. (a) insulin deficiency and the need for exogenous insulin therapy and (b) the presence of islet-directed autoantibodies. For patients who develop DM over the age of 20 years, these criteria should be supplemented with a genetic risk score and the presence of certain immunological markers [98]. Classification can also be aided by C-peptide measurements. The β-cell destruction associated with T1DM appears to proceed significantly faster in infants and children than in adults. However, the presence of pancreatic autoantibodies is one of the symptoms of T1DM; antibody negativity does not exclude T1DM, so it is necessary to avoid relying solely on “lean” or “young” phenotypes.

The ADA T1DM diagnostic criteria also propose measurements of pancreatic autoantibodies [99], as approximately 5–10% of European new-onset T1DM patients are negative for islet antibodies. While this absence does not exclude T1DM, it does prompt further consideration of diagnosis [100,101]. Autoimmune T1DM manifests itself as the autoimmune destruction of pancreatic β-cells and accounts for 5–10% of all diagnoses of diabetes. The destruction involves islet cell autoantibodies and autoantibodies to glutamic acid decarboxylase (GADA65), insulin (IAA), the islet antigen 2 (IA-2) and IA-2B tyrosine phosphatases, zinc transporter 8 (ZnT8), and tetraspanin-7 (Tspan7) [6,85,98]. At least one of these autoantibodies is detected in 85–90% of patients with T1DM in whom fasting hyperglycemia is initially detected [7].

According to a recent model, the progression of T1DM consists of three stages [102]. Stage 1 of T1DM, the presymptomatic stage, is characterized by the presence of two or more islet-directed autoantibodies and normoglycemia. IGT and impaired fasting glucose (IFG) are not detected, and HbA1c is normal. At this stage, the five-year risk of developing T1DM is ~44% [6]. Stage 2, also presymptomatic, is characterized by the presence of islet autoantibodies, usually multiple, and dysglycemia. While dysglycemia is associated with inadequate insulin secretion following a glucose or nutrient challenge, it is not yet diagnostic of DM at this stage. Other criteria include IFG (FPG 100–125 mg/dL (5.6–6.9 mmol/L)), IGT (2 h plasma glucose (PG) 140–199 mg/dL (7.8–11.0 mmol/L)), HbA1c 5.7–6.4% (39–47 mmol/mol), or ≥10% increase in HbA1c. At the time of progression to Stage 2, the risk of developing Stage 3 T1DM is ~60% within two years and ~75% within five years [6,98]. In Stage 3, the symptomatic stage, T1DM is usually clinically diagnosed, and symptoms are usually present. Autoantibodies may become absent, and overt hyperglycemia is observed. T1DM is usually diagnosed by standard criteria. Insulin therapy may be initiated [6,98].

Low C-peptide concentrations indictive of severe endogenous insulin deficiency may also be observed in Stage 3. Their concentrations may be useful markers for assessing DM for three years after clinical diagnosis and can be used to guide therapy [103,104]. Combining measurements of C-peptide and blood glucose levels in a sample provides an estimate of endogenous insulin production. These measurements are particularly valuable in chronic disease, when levels fall below 300 pmol/L [103,105]. At presentation, C-peptide levels in T1DM are higher, making it more difficult to distinguish from T2DM, in which levels typically remain above 600 pmol/L. As C-peptide thresholds cannot be categorically defined, C-peptide must be interpreted with other clinical and laboratory features [25]. Classification is also complicated by the fact that at the time of clinical diagnosis in Stage 3, serum and urinary C-peptide levels are lower in children than in adults [106,107]. It has been suggested that patients with a random C-peptide level ≤ 300 pmol/L should be managed mainly with insulin, while insulin should be combined with other DM therapies in patients with higher C-peptide levels (> 300 pmol/L) [25].

##### Risk Factors for T1DM

Type 1 diabetes is a complex autoimmune disorder associated with multiple genetic, immune, and environmental factors involved in its pathophysiology [108].

- Genetic risk factors for T1DM

Only 10–15% of patients with T1DM have a first- or second-degree relative with the disease, and as such, type 1 is usually diagnosed in patients without a family history. The genetic susceptibility to T1DM can be summarized as follows: identical twins have a concordance of 60–70% [109], siblings 6–7%, and children with a parent with DM 1–9% [110]. In contrast, the prevalence in the general population is 0.4% [111]. In the case of T2DM, a twofold to sevenfold risk exists between first-degree relatives. In male relatives, this risk is higher 80% and in female relatives is higher 230% in the case of diabetic mothers of diabetic probands [91]. T1DM is slightly more common in men and boys than in women and girls [84].

The main genes involved in the development of T1DM lie within the major histocompatibility complex (MHC) region, also called the human leukocyte antigen (HLA), located on human chromosome 6 [112]. Approximately 50% of genetic risk is associated with class II HLA alleles, which encode DR and DQ; these are the strongest determinants, with DR3-DQ8 and DR4-DQ8 present in the majority of patients with T1DM [113]. More than 90% of the patients with T1DM have HLA-DR4 DQB1*0201 (also referred as DR3-DQ2) or HLA-DR4 DQB1*0302 (also referred as DR4-DQ8) haplotypes, with about 30% of the patients having both haplotypes (DR3/4 heterozygotes). In contrast, these haplotypes are present in 40% of healthy subjects. Other DR4 alleles, such as DRB1*0403 and DRB1*0402, reduce the risk of T1DM, even in the presence of the high-risk allele DQB1*0302. The HLA allele DQB1*0602 also protects against T1DM development. This allele is detected in about 20% of the general population but in only 1% of children who have developed T1DM [112].

It has also been proposed that 15% of the genetic predisposition is contributed by non-HLA-genes, with more than 60 other non-HLA-gene loci being associated with T1DM [86]. These include the insulin gene (*INS*), located on human chromosome 11, the cytotoxic T-lymphocyte-associated antigen-4 gene (*CTLA-4*), located on human chromosome 2q33, and the genes associated with immune regulation, such as the protein tyrosine phosphatase non-receptor type 22 (*PTPN22*) gene, located on human chromosome 1, the interleukin-2 receptor (*IR2RA*) gene, also known as CD25, and the protein tyrosine phosphatase non-receptor 2 (*PTPN2*) gene, located on chromosome 18p11.21. Some of these genes are associated with other autoimmune diseases, such as thyroiditis and rheumatoid arthritis [86]. These genes include combinations of 67 single nucleotide polymorphisms (SNPs) (14DR-DQ, 21 other HLA and 32 non-HLA) and variable number of tandem repeat (VNTR) sequences. These genes contribute to the estimated 15% of the non-HLD genetic predisposition [112].

Insulin-VNTR is associated with polymorphisms in the promoter of insulin gene. It is believed to account for about 10% of the genetic predisposition for T1DM. Two common classes are known: small class (26–63 repeats, type I) and large class (140–243 repeats, type III) [114]. The polymorphisms alter the amount of mRNA in the thymus, affecting immune tolerance to insulin [86]. Type III is present in approximately 30% of the gene population and is believed to protect against T1DM, regardless of the individual HLA haplotypes. The Type III class is believed to express its protective role by negative selection and deletion of insulin-reactive T lymphocytes, which are involved in the autoimmune destruction of β-cells [114,115].

The *CTLA-4* gene is also associated with T1DM. It plays a suppressive role by transmitting a negative signal and inducing energy in activated T lymphocytes [116]. A naïve T lymphocyte requires a triple complex to identify the antigen: the antigen (Ag), the HLA molecule of the antigen presenting cell (APC), and the T-lymphocyte antigen receptor (TCR). Unfortunately, this complex is not itself capable of stimulating the naïve T lymphocyte to proliferate and differentiate into an active form. This process requires the presence of costimulatory molecules, such as B7.1 (CD80) and B7.2 (CD86) glycoproteins on the APCs and the CD28 receptor on T lymphocytes. After activation, the T lymphocyte expresses the CTLA-4 receptor: the sequence of the expressed receptor resembles that of CD28 but has 20-fold greater affinity for B7 glycoproteins. After the CTLA-4 receptor binds to the B7.1 or B7.2 molecules of the APC, it transmits negative signals to the T lymphocyte; these limit the production of interleukin-2 (IL-2) and the proliferation of stimulated lymphocytes, thus avoiding the intense stimulation and proliferation of T lymphocytes. In autoimmune diseases, such as T1DM, the patient is more likely to possess *CTLA-4* polymorphisms, which decrease the intracellular expression of the protein and allow excessive stimulation and proliferation of T lymphocytes. This results in uncontrolled progression of immune response and autoimmune imbalance [116,117].

Of particular interest is the polymorphism C1858T (Arg620Trp), found in the *PTPN22* gene; this gene codes protein tyrosine phosphatase non-receptor type 22, also known as lymphoid-specific tyrosine phosphatase (LYP), which inhibits the activation of naïve T lymphocytes by the dephosphorylation and inactivation of the tyrosine-protein kinase CSK. A missense mutation in the *PTPN22* gene is associated with several autoimmune diseases, including T1DM. The mutation decreases the binding affinity of LYP to CSK, resulting in uncontrolled T-cell activation and an autoimmune reaction [118,119]. The presence of C1858T increases the risk of T1DM up to five times in homozygous carriers and two times in heterozygous carriers [91].

The AIRE protein (autoimmune regulator) is encoded by the *AIRE* gene located on the chromosome 21q22.3. This protein is a transcription factor expressed mainly in the epithelial and dendritic cells of the thymus marrow, where it is involved in the regulation of autoimmunity. Mutations are often observed in this gene in patients with T1DM, suggesting that the AIRE protein plays a part in the expression and presentation of autoantigens against insulin, GADA, and IA-2 in the thymus [120,121].

The interferon-induced helicase C domain 1 (*IFIH1*) gene, located on chromosome 2q24.2, is expressed in β-cells. The gene encodes MDA5 (melanoma differentiation-associated) RNA, activating a cascade of anti-viral immune responses. However, while the gene is associated with susceptibility to T1DM, some rare variants are believed to play a protective role due to lost or reduced protein expression. Despite this, commonly detected *IFIH1* SNPs are associated with the development of T1DM [122,123].

- Environmental risk factors for T1DM

Some environmental risk factors have been associated with the development of T1DM. These include some viral infections and the introduction of gluten-containing solid foods during the first three months of life, which may play a part in the development of islet autoantibodies.

(a) Viral infections

Some viruses, such as mumps and Coxsackie B, may damage pancreatic β-cells. While the role of viruses in T1DM development remains uncertain [91], retrovirus infection often precedes the development of T1DM, and vaccination against rotavirus decreases the risk of diabetes [91]. Some evidence suggests that adenovirus and mumps virus may also be involved in the development of T1DM, although infection by these viruses is uncommon. Enteroviruses may be involved in the early phase of the development of T1DM, causing the activation of innate immunity [124,125]. There are also observations suggested protective role against T1DM [126]. In addition, children exposed to rubella during pregnancy were also found to be at increased risk of T1DM [127]. Coxsackie virus infection may also increase T-cell autoactivation, resulting in inflammation, tissue damage, and the release of islet autoantibodies [128]. Other studies have implicated inter alia enteroviruses, cytomegalovirus, Ljungan virus, and herpes virus in T1DM development [129,130]. While it has been proposed that vaccination may play a part in the development of autoimmune diseases, including T1DM, this has not been supported by evidence [131].

(b) Dietary factors

The association between diet and the development of T1DM remains controversial. Several studies suggest that the early introduction of milk into the diet might support the development of T1DM, possibly due to exposure of infants to insulin contained in the milk, thus triggering an autoimmune response [132]. Children unable to develop oral tolerance to bovine insulin were found to have an increased risk of β-cell autoimmunity; this suggests that the reaction of the immune system to bovine insulin may drive the production of antibodies that may attack human insulin [133,134]. It is also suggested that a component of the albumin from cow’s milk, called ABBOS (17 amino-acid peptide, positions 152–167), may act as a self-reactive epitope because it is similar to the p69 protein present on the surface of β-cells [135,136].

An observational study of a large prospective birth cohort in European countries found the early introduction of gluten-containing foods, e.g., cereal, in infant diets, i.e., during the first three months of life, to be associated with a significant increase in islet autoantibody production [137]. The findings indicate higher numbers of islet autoantibodies to be present in at-risk children, with the increases correlating with both the dosage and the timing of gluten intake. Similar results were also obtained in another study [138]. It is suggested that the intestinal inflammation and T-cell activation caused by gluten may contribute to the development of β-cell autoimmunity [139].

Many other risk factors have been associated with T1DM, such as gut microbiota reduction [112], psychosocial factors [91], lack of Vitamin D, and early exposure to toxins (e.g., Streptomyces-infected root vegetables) [125].

##### Complications and Comorbidities of T1DM

T1DM is a chronic disorder in childhood, and early diagnosis is essential for its prevention; if left untreated, several complications and comorbidities may develop. Potentially life-threatening complications associated with T1DM are hyperglycemia and ketoacidosis. Hypoglycemia may also have adverse effects on cognitive function [140], and hypoglycemic events are associated with 4 to 10% of T1DM-related deaths [141,142]. Most cases of hyperglycemia are reported in preschool and young primary school children. It has been suggested that this may be due to fasting, improved emotional instability, or insulin overdose [91]. Also, in the case of adult patients, fear of hyperglycemia remains a major problem in the clinical management of T1DM [143], which can have a strong influence on quality of life and glycemic control in this group [95].

Nearly 30% of younger patients, i.e., aged under 20 years, with newly diagnosed T1DM are diagnosed with DKA [92]; DKA also accounts for 13–19% of T1DM-related mortality [93]. The prevalence of DKA among adult patients with T1DM is unknown [95], although it is more common among women than men and among people with higher HbA1c [93]. Predisposing factors are thought to include poor metabolic control, certain psychosocial factors, puberty, insulin omission, pump failure, or treatment error [91,95]. For adult patients with T1DM, the primary risk factors are non-compliance and infections [144].

The microvascular complications of T1DM are mostly associated with retinopathy, neuropathy, and nephropathy [86,93], although they also affect cognitive function, the heart, and other organs following damage to the capillaries caused by high blood glucose levels. Such changes are particularly apparent in the neurons, kidneys, and retina, where glucose uptake is independent of insulin [125]. The primary risk factor for microvascular complications is hyperglycemia; therefore, reducing HbA1c through intensive T1DM management has been found to reduce their incidence by about 70% and slow their progression [93]. Up to 50% of patients with T1DM are at risk of neuropathy. Damage to the macula or retina, or both, may also occur during DM, resulting in visual problems and blindness, with the risk of retinopathy increasing with disease duration. Many of the ophthalmological and renal complications experienced during DM have been attributed to the microvascular changes associated with hyperglycemia, such as small vessel basement membrane thickening and increased endothelial cell permeability [145].

Patients with T1DM are also at increased risk of macrovascular complications, i.e., those associated with large blood vessels, such as atherosclerosis and thrombosis of the heart, peripheral arteries, and brain [93]. Patients with T1DM are at four times greater risk of cardiovascular disease than non-diabetic individuals. This increased risk of macrovascular complications is associated with inter alia blood pressure, lipid and glucose metabolism, and various genetic factors, while hyperglycemia is also associated with a greater chance of cardiovascular disease. Worryingly, patients with T1DM are 30% more likely to exhibit hypertension than non-diabetic individuals, which significantly increases the likelihood of coronary artery disease and stroke. Cardiovascular disease is a major cause of premature morbidity and mortality, and the life expectancy of patients with T1DM is eight to thirteen years shorter than that of healthy subjects [146,147]. The pathogenesis of micro- and macrovascular complications is believed to be associated with the direct and indirect effects of advanced glycation end products (AGEs), activation of protein kinase C (PKC), and defects in polyol metabolism [125].

Hyperglycemia is also believed to play a key role in the development of neuropathy by damaging the sensory, autonomic, and motor neurons of the peripheral nerves. Such damage can result in axonal thickening and loss, microfilament loss, neural demyelination, and neuron death [145].

In 30–40% of patients with T1DM, diabetic nephropathy results in the reduction, or complete loss, of glomerular filtration and albuminuria (> 300 mg/day) [148]. The main metabolic risk factors of diabetic nephropathy include hyperglycemia, hypertension, and hyperlipidemia [145].

##### Subtypes of T1DM

Type 1 diabetes exists as two subtypes: idiopathic diabetes mellitus (IDM) and fulminant type 1 diabetes mellitus (FT1DM).

- Idiopathic diabetes mellitus

The first identification of idiopathic diabetes mellitus (IDM) was made in 1984 by Ahren and Corrigan, who described a subgroup of patients with DM in Tanzania; the patients demonstrated varying need for insulin therapy over time and developed transient ketoacidosis [149]. Later, an unusual type of DM termed *atypical diabetes* was reported [150] among 12 young African Americans with obesity who required medical care due to spontaneous DKA. Interestingly, none of these patients possessed anti-islet cell antibodies (ICA), and all had a strong family history of T2DM. The type was also associated with a specific set of symptoms: despite presenting with the phenotypic characteristics of T2DM, the patients exhibited ketoacidosis, characteristic of T1DM. However, further observations indicated that feature to be more consistent with T2DM than with T1DM. Hence, IDM is classified as a clinically distinct type of T1DM. In 1997, the ADA proposed two subcategories for T1DM: classical autoimmune T1DM (ADM) and IDM [151].

Idiopathic diabetes mellitus (IDM) is a heterogeneous autoantibody-negative insulin-deficient form of DM, which is more common in African American and Asian patients; however, it has also been described in Native Americans and Hispanic Americans, as well as in European Mediterranean patients [3,152]. The genetic and/or clinical heterogeneity of IDM have been examined in a number of studies and cases. One study reclassified a group of 290 selected case reports, cross-sectional studies, and cohort studies according to ADA criteria, such as autoantibody negative, onset <35 years old, nonobese, and not HLA-associated types [153]. After reclassification, 201 cases were identified as IDM, 30% of which were autoantibody-negative. These were linked to monogenic diabetes, such as maturity-onset diabetes of the young (MODY) and demonstrated mutations in the INS, KCNJ11, and KLF1 genes, associated with neonatal diabetes. The authors recommend the use of advanced genetic screening for improving diagnosis and management [153].

A prospective study in Qatar diagnosed T1DM in 1157 DM patients. Among these, 63 were antibody-negative. Genome sequencing revealed that four of these patients had MODY, two had Wolfram syndrome, one had H syndrome, and three had variants of uncertain significance in MODY genes. The most common age of IDM diagnosis was 10–14 years. The C-peptide level was low in 30 patients (56.6%) and normal in 23 (43,4%). The mean BMI of these patients was normal, and 33% of the IDM patients had a history of DKA. The analysis found DKA to be less common in patients with IDM compared to those with other types of T1DM. The authors conclude that based on the presence of the detected mutations in MODY patients, autoantibody testing and genetic screening are necessary to identify causes of monogenic DM in IDM [154].

In another cross-sectional study, 23.8% of all newly diagnosed patients with T1DM were found to have IDM. The IDM patients were characterized by an older onset age, higher BMI among adults, lower HbA1c, higher levels of fasting C-peptide, and 2 h postprandial C-peptide; they were also highly likely to have a family history of T2DM and to carry an O-susceptible HLA haplotype compared to ADM patients. In addition, patients who had overweight, had a family history of T2DM, and lacked susceptible HLA haplotypes were also more likely to be negative for autoantibodies [155].

A retrospective analysis found patients with IDM to have a significantly higher BMI, waist circumference (WC), Visceral Adiposity Index (VAI), LDL-cholesterol, glutamic oxaloacetic (GOT), and glutamic pyruvic (GPT) transaminases compared to patients with ADM. The patients with IDM also demonstrated a higher prevalence of hepatic steatosis, visceral obesity, and hypercholesterolemia than the ADM patients and a higher cardiometabolic risk [156].

In 2014, Babiker et al. [157] described idiopathic remitting diabetes in a female Caucasian patient. The patient was diagnosed aged 15 years with symptoms of diabetes and was treated with basal bolus insulin. Insulin therapy was discounted two years after diagnosis due to hypoglycemia. During pregnancy, 13 years after diagnosis, she obtained a normal oral glucose tolerance test and HbA1c. At the time of the study, she appeared to be free of T1DM and T2DM [157]. Another case of idiopathic remitting T1DM was described in a 17-year-old male Caucasian patient with polyuria and polydipsia. T1DM was diagnosed on the basis of a formal laboratory glucose and HbA1c. A basal-bolus insulin regimen was commenced. From 21 months after diagnosis, the insulin requirements consistently fell. Insulin therapy was stopped at 28 months due to an increased frequency of hypoglycemic events [158].

Idiopathic remitting diabetes is rare and was identified in a single case from a database of >15,000 patients. Remitting diabetes of a similar pattern has been described previously; however, the only identified case [157] was a report describing two boys with idiopathic remitting diabetes [159].

The pathophysiologic mechanism(s) associated with the etiology of IDM is unknown. As such, further intensive cohort studies are needed to better describe the natural history and clinical course of IDM [160].

- Fulminant type 1 diabetes mellitus

Fulminant type 1 diabetes mellitus (FT1DM) can be subdivided into autoimmune (Type 1A) and idiopathic types (Type 1B, IDM, and T1B) and is classified as T1B by the ADA and WHO [161,162]. The condition is characterized by markedly rapid pancreatic β-cell destruction with aggressive progression of hyperglycemia and ketoacidosis [163,164]. Most cases of FT1DM have been diagnosed in East Asia, particularly Japan [165]: the frequency of FT1DM was found to be 19.4% in Japan, 7.1% in South Korea, and 1.5–5.5% in China [165,166]. Cases of FT1DM have also been reported in other Asian countries and regions, including the Philippines, Taiwan, Malaysia, Vietnam, Thailand, India, and Laos, and in Australia [166]. While cases have been noted in Caucasian populations [167,168], case reports and cohort studies indicate that FT1DM is rare in Western countries: three cases have been reported in France, one case in Spain, and one Italian patient in an IDM group, who was negative for GADA antibodies [166]. A more detailed analysis identified 5000 to 7000 patients with FT1DM in Japan in 2009. Data from the Japanese Ministry of Health, Labor, and Welfare estimate around ten million cases of DM between 2016 and 2017, of which approximately 120,000 had T1DM; therefore, this group may include approximately 10,000 patients with FT1DM [165].

ADM is more common among women; however, the incidence of FT1DM does not appear to be influenced by sex. More than 90% of patients with FT1DM have adult onset, and the mean age of onset is higher than for ADM [169].

Based on the *Criteria for the definite diagnosis of fulminant type 1 diabetes mellitus (2012)* [170], FT1DM is confirmed by the following criteria: (1) occurrence of diabetic ketosis or DKA soon after (within approximately 7 days) the onset of hyperglycemia; (2) plasma glucose levels ≥160 mmol/L (≥288 mg/dL) and HbA1c level <8.7% at the first visit; and (3) urinary C-peptide excretion <10 mg/day or fasting serum C-peptide levels <0.3 mg/mL (<0.10 mmol/L) [171].

The onset of FT1DM is rapid. The disease is characterized by rapid pancreatic β-cell destruction, resulting in the almost complete loss of islet β-cell function within a short period. This is followed by the complete loss of insulin secretion and the swift progression of hyperglycemia and DKA. While pancreatic enzyme levels are elevated, no pancreatic autoantibodies are observed, including glutamic acid decarboxylase antibodies, the serum marker used for the diagnosis of ADM. It is also suggested that both islets and pancreatic acinar cells are destroyed in a different manner to ADM [165,172,173,174].

Several papers have described cases in which FT1DM was associated with pancreatitis or myocarditis. The first description of FT1DM accompanied by both acute pancreatitis and myocarditis was presented in 2020. The authors suggest that the two with the diseases share a common etiology that is associated with the pathophysiology of FT1DM [175]. The development of FT1DM can be also triggered by pregnancy [176]; this pregnancy-associated-FT1DM (PFT1DM) typically occurs within two weeks after delivery [162]. According to analyses performed in Japan, PFT1DM accounts for around 21% of all cases of FT1DM and is associated with intrauterine fetal death in approximately 67% of cases [162].

Investigations based on candidate gene analysis indicate that HLA gene class II is a genetic factor in FT1DM. A large proportion (41.8%) of Japanese patients with FT1DM have the HLA class II haplotype DR4-DQ4 [177]. Individuals with DR4-DQ4 encoded by DRB1*04:05-DQB1*04:01 and DR9-DQ3 encoded by DRB1*09:01-DQB1*03:03 [178] are susceptible to the disease [166]. These haplotypes are similar to those associated with T1DM reported in the Japanese population and appear to be unique to Asian populations, being rare among Caucasian populations [179]. Haplotype DRB1*15:02-DQB1*06:01 is associated with strong resistance to T1DM and has no effect on FT1DM [180]. Investigations of class II HLA in Korean patients found DRB1*04:05-DQB1*04:01 to be associated with a higher risk of FT1DM, showing that DRB1*15:02-DQB1*06:01 exhibits no significant resistance in FT1DM [181]. In the Chinese population, haplotypes DQA1*03:03-DQB1*04:01 and DQA1*01:02-DQB1*06:01 appear to be more frequent in patients with FT1DM than in those with ADM [182]. This is described in more detail elsewhere [165,166,172,183].

Viral infection may also be an initial factor in the pathogenesis of FT1DM. For example, a strong correlation was observed between the onset of FT1DM and viral infection in the pancreas. Flu-like symptoms were observed in 71.7% of patients with FT1DM, while these symptoms are less frequently observed in patients with ADM [169,184]. Among the infections associated with development of FT1DM are those by enterovirus, rotavirus, cytomegalovirus, Epstein–Barr virus, human herpesvirus G, and echovirus [184,185,186,187]. FT1DM was also reported after SARS-CoV-2 infection [188]. It has been suggested that viral infections induce pro-inflammatory responses, affecting lymphocytes and antigen-presenting cells, and these might play an important part in the adaptive immune response to pancreatic β-cells [189].

#### 2.1.3. Gestational Diabetes Mellitus

Gestational diabetes mellitus (GDM), defined as “glucose intolerance of various degrees that is first detected during pregnancy”, represents one of the most common complications of pregnancy. It has long been known that the occurrence of DM before pregnancy can have a negative influence on fetal and neonatal outcomes [190]. Studies in the 1940s suggested that a diagnosis of DM during pregnancy was associated with higher fetal and neonatal mortality [191]. In 1957, the term *gestational diabetes* was used to indicate a transient condition associated with fetal outcomes adversely, which abated after delivery [192]. Subsequent studies later found the degree of glucose intolerance during pregnancy to influence the risk of developing DM after pregnancy [193]. The following years saw the development of new criteria for the interpretation of OGTTs, modern methods for measuring glucose, and a new definition of GDM as “glucose intolerance with onset or first recognition during pregnancy” [194,195].

According to WHO criteria, GDM is suggested by fasting plasma glucose ≥ 5.1 mmol/L, 1 h ≥ 10 mmol/L, and 2 h ≥ 8.5 mmol/L during a 75 g oral glucose tolerance test screening at 24–28 weeks or early screening for high-risk pregnancies. For non-pregnant adults, the criteria are HbA1c ≥ 6.5%, FPG ≥ 7.0 mmol/L, OGTT 2 h ≥ 11.1 mmol/L, or random ≥ 11.1 mmol/L plus symptoms (any one suffices). The steady growth in its prevalence has been attributed to the rise in obesity among women, the increasing age of pregnancy, the increased occurrence of DM in general, and better recording and testing regimens during pregnancy [196,197]. In 2013, a number of countries adopted a new set of International Association of the Diabetes and Pregnancy Study Group (IADPSG) diagnostic criteria and testing regimens for GDM. As such, a diagnosis of GDM may vary between countries with different definitions [196]; it has been suggested that implementing this new IADPSG definition could result in a two- to threefold increase in the diagnosis of GDM [198]. Even so, GDM is diagnosed in 14% of pregnant women worldwide, representing approximately 20 million births/year [199].

##### Prevalence of GDM

The prevalence of GDM depends on race, population type, age, body composition, and diagnostic criteria [200,201]. Worldwide, its prevalence ranges from 5% to 25%, with more than 90% of cases being diagnosed in South and Southeast Asia [202]. The Middle East and North Africa have the highest prevalence, at 27.6%. In Qatar, the prevalence of GDM in 2018 was 23.5% and increased to 31.6% from 2019 to 2022 [203]. Its prevalence in the United States increased from 1.2% in 1976 to 2.3% in 2010 and from 6.8 in 2016 to 8.3 in 2021 [201]. Some observations suggest a 23-fold increase to 9.2% in the US between 1979 and 2010 [204]. In Korea, GDM affects over 10% of all pregnant women [205].

Also, 30–70% of pregnant women with GDM have been found to demonstrate early gestational diabetes, i.e., hyperglycemia from <20 weeks of gestation. Such cases have worse pregnancy outcomes compared to those of late gestational diabetes, i.e., hyperglycemia from 24 to 28 weeks of gestation [206]. The rate of GDM may also be associated with BMI: during pregnancy, GDM was detected in 15% of women with overweight, 15% of women with obesity, and 21% of women with morbid obesity [207]. An increased risk of GDM is also associated with older age, elevated triglyceride levels, lower HDL cholesterol, and insufficient physical activity [208].

##### Complications and Comorbidities of GDM

The presence of GDM can increase the risk of maternal and neonatal complications.

- Health complications for mother

Where present, GDM can increase the likelihood of various short-term and long-term health complications. The short-term effects include increased risk of pregnancy hypertension, pre-eclampsia, cesarean birth, amniotic fluid excess, preterm membrane rupture, and ketoacidosis [200,209]. The mother is also at increased risk of shoulder dystocia [210]. The long-term effects include CVD, metabolic syndrome, and T2DM [211,212,213]. Apart from the stress of normal pregnancy, GDM is also associated with antenatal depression [214], postnatal depression, and postnatal stress disorder [215].

The initial symptoms of GDM are detected during the second or third trimester [200]. The presence of GDM during pregnancy can be associated with increased risk of periodontitis [216,217] due to the formation of a unique oral microbiota characterized by a pro-inflammatory dysbiosis; these conditions favor the development of bacteria that stimulate periodontitis and inhibit those that promote periodontal health [218].

Women with GDM diagnosed at 24–28 weeks of gestation also demonstrate reduced breastfeeding [219]; they also exhibit an increased risk of developing T2DM or prediabetes after childbirth. In addition, in the US, 40–60% of women with GDM were found to exhibit postpartum DM, 30% developed DM within the first year after delivery, and 5% were diagnosed each subsequent year [220,221]. In women with GDM, the risk of developing postpartum DM is 7–10 times higher as compared to women without GDM [220,222,223].

It has been proposed that GDM permanently alters the vasculature, increasing the risk of CVD by 63%, which may be partly explained by the increased BMI associated with GDM [224]. Hence, pregnant women with GDM have a higher risk of inter alia myocardial infraction and stroke compared to those without a history of GDM [225]. Women with GDM also demonstrate increased arterial stiffness and blood pressure [226] and a higher risk of metabolic syndromes, such as hyperglycemia and hypertriglyceridemia; elevated triglyceride levels cause hormonal changes, which can damage the lipid metabolism [227]. The condition is also associated with IR and glucose metabolism disorders, which may be observed after delivery [228]. During the early postpartum period, approximately 22–30% of women with a history of GDM have impaired glucose tolerance [229,230]. Many also experience changes in protein metabolism due to GDM [231].

Women with GDM are also at a significantly higher risk of ophthalmic morbidity, such as glaucoma, diabetic retinopathy, and retinal detachment, compared to those with no GDM during pregnancy [232]. It has also been suggested that GDM is associated with renal disease, the most common types being hypertensive renal disease without renal failure, chronic renal failure, and end-stage renal disease [233]. Women with a history of GDM are also at higher risk of the development of malignancies, such as ovarian, endometrial, and breast cancers [234,235,236]. They are also more likely to be diagnosed with pancreatic cancer [236,237].

- Fetal and neonatal complications

GDM also may have short- and long-term consequences for the infant.

- *Short-term neonatal outcomes*

Increased placental transport of substrates, such as glucose, amino acids, and fatty acids, stimulate the production of insulin and insulin-like growth factor 1 (IGF-1). These factors can cause fetal overgrowth, resulting in macrosomia at birth [238]. Macrosomia is believed to occur in as many as 50% of pregnancies in women with GDM, and such pregnancies are 10 times more likely to result in the delivery of an infant with macrosomia compared to a physiological pregnancy [239]. Deliveries of large-for-gestational-age (LGA) infants can be associated with an increased risk of shoulder dystocia, birth trauma, and cesarean section. The occurrence of GDM has also been associated with increased fat mass and disproportionate growth in the infant [240]. The chance of macrosomia increases from 9% in women with a mean blood glucose level <110 mg/dL to 34% in those higher blood glucose levels [241].

Neonatal hypoglycemia in newborns is indicated by a blood glucose level < 35–40 mg/dL during the first 12 h of life. The condition has been associated with hyperinsulinemia and is a common complication in macrosomic newborns [242].

Women with GDM are also at a higher risk of stillbirth than those with normal glucose levels, which may be related to the presence of chronic intrauterine hypoxia [239]. One study found 27% of infants born from mothers with GDM to exhibit perinatal asphyxia [243]; indeed, such infants are believed to be at an increased risk of intrauterine or perinatal asphyxia due to macrosomia. A cohort study performed in France found that infants have a 30% increased likelihood of perinatal death if born to mothers with GDM [244,245].

Hypocalcemia, i.e., the total concentration of calcium in serum <7 mg/dL, has been identified in between 5 and 30% of infants of mothers with GDM. The condition is typically asymptomatic and does not require treatment; however, the symptomatic form can cause jitteriness lethargy, apnea, tachypnea, and seizures [239].

Hypomagnesemia, i.e., serum magnesium < 1.5mg/dL, occurs in up to 40% of infants of diabetic mothers. It is not treated because it is usually transient and asymptomatic [240].

Hyperbilirubinemia is detected in 38% of infants of mothers with GDM, especially in preterm infants. It has been associated with macrosomia and polycythemia [239]. In pregnancies where the mother has GDM, the infant is at an increased risk of transient hypertrophic cardiomyopathy. The most common pathology is thickening of the inter ventricular septum, causing obstruction of left ventricular outflow; this may be due to fetal hyperinsulinemia [197,243].

- *Long-term neonatal outcomes*

Gestational diabetes has also been associated with long-term complications in the infant. Approximately 47% of younger people exposed to maternal DM during pregnancy were found to develop T2DM, compared to 6% of cases in children from mothers without DM [197,246]. It has been proposed that GDM in pregnant women may result in an increased risk of overweight and obesity. These outcomes may be due to fetal hyperinsulinemia, which impairs the development of adipose tissue and pancreatic β-cells. Obesity was recorded in 1.8% of children from non-GDM mothers, while increased BMI was noted in 4.9% of children of GDM mothers and 7.8% of children whose mothers had GDM uncontrolled by diet [240,247].

Cardiometabolic outcomes occur in 19% of infants of mothers with GDM, which may be associated with functional and structural changes of the fetal heart [248]. Gestational diabetes may be also associated with neurodevelopmental outcomes in children; for example, GDM has been related to autistic spectrum disorder (ASD) and attention-deficit/hyperactivity disorder in children [249,250,251]. This subject has been examined elsewhere in more detail [197,201,213,231,240,245,252,253,254].

- Pathophysiology of GDM

The pathophysiology of GDM is attributed to the spontaneous development of hyperglycemia during pregnancy. Usually, GDM is caused by a combination of β-cell dysfunction and chronic IR, with these being the main critical components of the pathophysiology of GDM. It has been postulated that the main pathophysiological mechanism behind hyperglycemia in GDM is damage to insulin secretion or relative insulin deficiency, which occurs in response to IR. The interaction of insulin deficiency and IR in determining glycemia can be described by the disposition index, which is specifically the product of insulin secretion and insulin sensitivity [195,231]. This index can be used to classify GDM as insulin-resistant, insulin-deficient, or mixed, i.e., GDM with both causes. Of these, insulin-resistant GDM is the most common form (50–60% of women with GDM), and insulin-deficient GDM is the least (15–30% of women with GDM) [231].

Pregnancy is associated with an increase in insulin resistance (IR), which has been attributed to increases in various local and placental hormones, such as estrogen, progesterone, leptin, cortisol, placental lactogen, and placental growth hormone. These increases are particularly notable in the second and third trimesters of pregnancy, resulting in a negative effect on insulin activity [229].

- *β-cell dysfunction*

The primary role of pancreatic β-cells is to store and secrete insulin in response to the glucose level. β-cell dysfunction impairs their ability to adequately sense the plasma glucose concentration or release sufficient insulin in response [197,253]. The mechanisms of islet β-cell dysfunction can be varied and complex [255,256]. Impairments can occur at any stage during the synthesis or release of insulin, such as inter alia during the synthesis of pro-insulin, post-translational modification, and sensing blood glucose concentrations [253]. These mechanisms are also determined by certain genes associated with GDM, such as *Kcnq1* and *GCK*, and by metabolic stress during pregnancy [257].

It has also been proposed that IR might be involved in islet β-cell dysfunction. Reduced glucose uptake, stimulated by insulin, causes hyperglycemia, which stimulates the β-cells to synthesize and release additional insulin in response. Pancreatic β-cells may also fail when exposed to high levels of glucose, known as glucotoxicity [258]. Hence, it appears that islet β-cell dysfunction is the first step in a vicious cycle of hyperglycemia IR and further dysfunction of pancreatic β-cells [253].

Animal studies indicate that glucose homeostasis also depends on the number of β-cells. Zucker fatty (ZF) rats, after 60% pancreatectomy, mostly recover β-cell mass by one-week post-surgery but still develop hyperglycemia. These results suggest that a larger short-term change in β-cell mass overburdens the remaining β-cells, reducing the glucose-stimulated secretion of insulin [259]. Further data from animal studies and some post-mortem human studies indicate that reduced β-cell hyperplasia [260], β-cell mass, β-cell number, and β-cell dysfunction are involved in GDM [253].

- *Chronic insulin resistance*

Insulin resistance is diagnosed when cells fail to adequately respond to insulin. The most common cause is a failure of the intracellular insulin signaling pathway [40]. This failure impairs the translocation of human glucose transporter 4 (GLUT4) from the intracellular compartment into the cell membrane: GLUT4 transports glucose from the blood into cells. In women with GDM, such transport is 54% lower in comparison with women without GDM [261]. Impaired insulin signaling also causes many cellular disturbances, such as impaired glucose, lipid, and protein metabolism and intracellular respiration, and inhibits various processes of differentiation, cell division, and apoptosis. These disturbances can have a pathogenic effect on embryogenesis [40].

- *Hormonal and immune dysfunction*

One factor in the pathogenesis of IR may be impaired hormonal function. Neurohormones play several roles, such as the regulation of appetite, active energy expenditure, and basal metabolic rate, which are made possible by a complex network of central and peripheral signals [262,263] regulated by the circadian clock. Among these regulators are adipokines, such leptin and adiponectin, which play a role as cell signaling proteins and are mainly secreted from adipose tissue [253].

Leptin is secreted by adipocytes in response to adequate energy stores. Its primary site of action is the neurons within the arcuate nucleus of the hypothalamus, where it decreases appetite and increase energy expenditure; this is achieved by inhibiting the appetite stimulators neuropeptide Y (NPY) and agouti-related peptide (AgRP) and activating the anorexigenic polypeptide proopiomelanocortin (POMC) [264]. While leptin was initially proposed as a potential therapy against obesity [265], the majority of obese subjects in trials were found to develop leptin resistance. Obesity is associated with excessive concentrations of plasma leptin, i.e., hyperleptinemia [266]. Leptin is also produced by the placenta, which represents the main source during pregnancy [267]. Placental production of leptin has been found to increase during GDM, probably caused by placental IR, resulting in hyperleptinemia; this facilitates the transport of amino acids across the placenta and may be involved in the development of macrosomia [268].

Adiponectin is primarily secreted by adipocytes, although plasma adiponectin levels are inversely proportional to adipose tissue mass: increased obesity is associated with decreased plasma adiponectin concentrations. Most significantly, decreased levels of adiponectin are associated with GDM [269], possibly due to its role in IR: it enhances the intracellular insulin signaling pathway and fatty acid oxidation but inhibits gluconeogenesis [270,271]. These actions are made possible by the activation of AMP-activated protein kinase (AMPK) within insulin-sensitive cells. In the liver, activated AMPK increases insulin receptor substrate 1 (IRS-1) activity caused by the activation of the transcription factor peroxisome proliferator-activated receptor alpha (PPARα). Adiponectin also stimulates insulin secretion by upregulating insulin gene expression and stimulating the exocytosis of insulin granules from β-cells [272]. It has hence been suggested that adiponectin can limit fetal growth by impairing the intracellular insulin signaling pathway and transport of amino acids across the placenta [253]. Interestingly, it has also been found that in addition to various cytokines, such as TNF-α, IL-6, and interferon gamma (IFN-γ), adiponectin can also be regulated by leptin, as observed in the syncytiotrophoblast of the placenta [273].

A successful pregnancy requires a complex and tightly regulated immune response to ensure the survival of the semi-allogenic fetus, which contains both maternal and paternal antigens [274]. This immunological balance is maintained by a shift from a Th1 (pro-inflammatory) to a Th2 (anti-inflammatory) cytokine profile, which reduces the likelihood of fetal rejection [275]. A crucial role in this process is played by regulatory T cells (Tregs), which suppress excessive maternal immune responses and promote an environment conducive to fetal tolerance [276]. In GDM, the balance may shift towards a pro-inflammatory state, exacerbating IR and dysregulating glucose metabolism [277]. Pregnant women with GDM present elevated levels of pro-inflammatory cytokines, such as TNF-α, IL-6, and interleukin-1β (IL-1β) [278,279], which are produced by activated maternal immune cells and the placenta. In addition to being involved in systemic inflammation, these cytokines directly influence the intracellular insulin signaling pathway [280]. For example, TNF-α inhibits the phosphorylation of IRS-1, impairing glucose transport into cells in peripheral tissues [281,282], and both IL-6 and IL-1β enhance the inflammatory cascade by disrupting the metabolic homeostasis [283].

A number of other factors, such as placental hormones and placental transport, genetic risk factors, adipose tissue inflammation, oxidative stress, and the gut microbiome, are also believed to be associated with GDM [197,205,253,277].

The most effective treatment for GDM is widely regarded to consist of insulin therapy and lifestyle intervention [197].

### 2.2. Rare Types of Diabetes Mellitus

#### 2.2.1. Latent Autoimmune Diabetes in Adults

Latent autoimmune diabetes in adults (LADA) is also referred to as diabetes mellitus type 1.5 (T1.5DM), slow-onset autoimmune diabetes (SAID) [284], slowly progressive insulin-dependent diabetes (SIDDM) [285], double diabetes [286], late type 1 diabetes, youth-onset diabetes of maturity, latent-onset type 1 diabetes, and antibody-positive non-insulin-dependent diabetes [287].

Based on its mixed clinical manifestations, it has been proposed that LADA represents an intermediate condition between T1DM and T2DM, as noted by the WHO in 2019 and the International Expert Panel in 2020 [5,104]. In 2021, Chinese experts proposed that LADA should be classified as a slowly autoimmune T1DM based on its etiology [288]. In 2022, the ADA proposed that LADA should be classified as a type of T1DM [289], despite being genetically distinct from T1DM and T2DM [290]. The term *LADA* was first introduced in 1993 [291], indicating it to be a distinct autoimmune subtype of DM. A multicenter study in Europe, Asia, and North America found that 4 to 14% of patients diagnosed with T2DM were also positive for diabetes-related autoantibodies [292,293], which adds further evidence that LADA should be regarded as a hybrid of T1DM and T2DM [294].

##### Prevalence of LADA

It is estimated that LADA accounts for approximately 2–12% of diabetic patients [104,295]. The prevalence of LADA varies between different countries and ethnicities. Recent studies suggest the existence of 10 million cases of LADA in the population of China [296,297]: the largest number of LADA patients in any single country [298]. A lower prevalence of LADA has been described in Italy (4.5%), Canada, the USA, and Europe (all 4.2%), Sardinia (5%), Korea (5.1%), and China (5.9%), while the highest prevalence was reported in Finland (9.3%), Bulgaria (10.1%), and the United Kingdom (13%) [299]. The prevalence of LADA also differs between northern and southern areas of Europe and China: higher levels have been reported in the northern region of Europe (7–14%) and in the northern region of China (6.5%) [300]. Due to its relatively high prevalence among patients with T2DM (4–14%) [292,293], LADA can be considered as the second most common type of DM after T2DM and the most common type of autoimmune DM in adults [299].

##### Clinical Features and Diagnosis

Clinically, LADA can be divided into an earlier insulin-dependent stage and a later insulin-independent stage [298,301]. The non-insulin-dependent stage responds to oral hypoglycemic drugs, and insulin therapy is not required for at least six months; this can help differentiate LADA from classic T1DM and ketone-prone T2DM. During this period, clinical features are similar to T2DM, *viz*. thirst, polyuria, and weight loss, and no clinical symptoms are detected [298,301]. Later, islet β-cell function deteriorates; diet, weight loss, exercise program, and conventional hypoglycemic drug therapies become ineffective, and insulin therapy becomes necessary: patients now enter the insulin-dependent stage [298,301].

Clinical presentation in LADA is less specific than T1DM or T2DM [302]. Like other forms of autoimmune DM, such as classical T1DM, LADA is characterized by the presence of specific diabetes-associated autoantibodies, such as IAA, GADA65, IA-2A, ZnT8A, and islet cell autoantibodies (ICAs) [301], Tspan7, and carboxypeptidase H (CPH) [303,304]. Most patients with LADA are positive for a single autoantibody, with the most predominant being GADA65 [305].

As the diagnosis of LADA is complex and lacks any clear, universally accepted criteria [306], it requires careful consideration of clinical and laboratory findings. Currently, the following criteria are proposed; however, none are categorized [104]: 1. adult-onset DM (age ≥ 30 years); 2. The presence of one or more diabetes-associated autoantibodies; and 3. no insulin requirement for at least 6 months after diagnosis [306,307].

While LADA shares some similar features with both T1DM and T2DM, some differences exist, which can be used to differentiate the conditions. T1DM is most often diagnosed in childhood or adolescence, its onset is acute, and β-cell destruction is rapid. In contrast, LADA is diagnosed in adulthood, and its course is slower. Patients with LADA retain endogenous insulin secretion for longer than those with T1DM. While insulin therapy is necessary at the diagnosis of T1DM, this therapy may be delayed for months to years after diagnosis in the case of LADA [307].

While LADA is often misdiagnosed as T2DM, some clear differentiating criteria exist. Patients with LADA are typically younger at diagnosis (<50 years) and have a low or normal BMI. It is also less likely that patients with LADA have IR or features of metabolic syndrome. These patients also have a higher prevalence of personal or family history of autoimmune disease. Other features characteristic of LADA, T1DM, and T2DM are given in Table 2 [307,308].

##### Pathophysiology of LADA

The pathophysiology of LADA is not fully clarified because the patients have genotype-characterized T1DM and the intermediate phenotype-characterized T1DM as well as T2DM [309,310]. Patients with LADA present some diabetes-associated antibodies (DAAs), as in patients with traditional T1DM. The detection of GADA suggests that the LADA can be divided into two subtypes: one without GADA autoantibodies, i.e., closer to T2DM, and one positive for GADA autoantibodies, i.e., closer to T1DM [311]. The initial GADA titer determines the persistence of autoimmunity, as well as the different degrees of LADA progression; it is also used to categorize LADA as either LADA-type 1 or LADA-type 2 [312,313].

Diabetes-associated antibodies play a significant role in the autoimmune destruction of islet β-cells, and for several years, islet β-cell autoimmunity was treated as the onset of LADA. It has been detected in nearly two-thirds of patients with LADA. Like T2DM, this destruction is followed by IR, which causes hyperglycemia. Therefore, the pathogenesis of LADA is associated with autoimmunity and metabolic disturbances due to IR [314]. LADA-type 1 is associated with a higher antibody titer, a greater degree β-cell function impairment, a higher insulin utilization rate, lower overweight and obesity rates, as well as a lower prevalence of metabolic syndrome. LADA-type 2 is characterized by a lower antibody titer; the metabolic phenotype, β-cell function, and overweight/obesity ratio are similar to those observed in patients with T2DM; and most cases of LADA-type 2 are associated with metabolic syndrome [315].

##### Association Between Genetic Factors and LADA

Evidence suggests that LADA is associated with many of the genetic features as in T1DM and T2DM. The primary risk for LADA is associated with the human leukocyte antigen (HLA) region, in particular HLA-DR and HLA-DQ; these are also characteristic of T1DM [309]. Most haplotypes associated with T1DM also confer LADA susceptibility [316].

The association of HLA haplotypes with LADA depends also on the population type. For example, the major risk factor for LADA is *DR9/DR9* in Chinese populations and *DR3/DR4* in Caucasian populations [317]. Currently, studies on the HLA genes associated with LADA are based on T1DM because the two share some common genetic susceptibility genes [290,318]. In Caucasian populations, the *DRB1/DQB1* genotype is associated with the age of onset: LADA patients with HLA *DRB1* and *DQB1* develop the disease at a younger age and have severe islet β-cell function [319]. *DQB1* genotypes are most often detected in patients with early-onset classic T1DM diagnosed before 20 years of age, followed by late-onset classic T1DM patients, diagnosed by after 35 years of age, and LADA patients [320]. Japanese patients with insulin-dependent LADA are more likely to present DRB1*0405-DQB1*0401, DRB1*0802-DQB1*0302, and DRB1*0901-DQB1*0303 haplotypes, whereas non-insulin-dependent LADA patients tend to exhibit only DRB1*0405-DQB1*0401 [321].

Some alleles are associated with T1DM protection, such as HLA DR2 and DQB1*0602 [322,323]. While children with these protective alleles are unlikely to develop T1DM [324], they confer less protection in adult-onset T1DM and LADA [325,326,327]. Also, HLA alleles involved in the development of T1DM, such as DRB1*03, and disease-protective alleles were detected at a similar frequency in LADA and classic adult-onset T1DM; this allowed the patient to be distinguished from T2DM [284].

Patients with LADA demonstrate a lower prevalence of DQB1*0302 and DQB1*0201/0302 and a higher frequency of DQB1*0602 than those with T1DM [328]. Also, DQB1*0302 and DQB1*0201/0302 appear to occur in patients with LADA at the same frequency as T1DM [329]. Several explanations have been proposed for this diversity [287]. The young patients with LADA carry high-risk alleles for T1DM, although their prevalence decreases with age [319]. It has also been noted that HLA-DQB1 and HLA-DRB are the strongest genetic factors associated with LADA: the highest risk is associated with the HLA haplotypes HLA-DRB1*04-DQB1*0302 (“DR4”) and HLA-DRB1*0301-DQB1*0201 (“DR3”) [330].

Other, non-HLA genes also play a role in LADA, although these genes are also associated with T1DM and T2DM. The strongest genetic factor associated with T2DM is transcription factor 7-like 2 (*TCF7L2*) [309], and the presence of a polymorphism in these genes may be involved in the development of LADA. For example, the variant rs7903146 is more frequently detected in patients with LADA than in patients with T2DM, especially in patients with low levels of GADA antibodies [104]. In addition, the rs7903146 and TCF7L2 rs12255372 polymorphisms are associated with LADA in patients with low GADA titers [331].

The protein tyrosine phosphatase non-receptor type 22 (*PTPN22*) gene is involved in the regulation of T-cell activation and immune responses. It is associated with T1DM and with LADA, especially with high GADA titers [104]. A polymorphism in this gene decreases the threshold for T-cell activation, causing heightened autoimmune responses and resulting in the progressive destruction of islet β-cells [306]. The insulin (*INS*) gene appears to have a stronger association with LADA characterized by high GADA titers and for LADA associated with multiple autoantibodies [309]. It promotes insulin production and influences immune tolerance, causing insulin to be presented as an autoantigen. The polymorphisms described in this gene are strongly associated with T1DM and influence the autoimmune destruction of pancreatic β-cells in LADA [306]. In addition, cytotoxic T-lymphocyte-associated antigen 4 (CTLA4), signal transducer and activator of transcription 4 (STAT4), interleukin 2 receptor alpha (IL2RA), and SH2B adapter protein (SH2B3) are T1DM-associated variants and T2DM-associated variants that also have a role in LADA [301,327,332].

##### Complications and Comorbidities of LADA

Hence, based on pathological and clinical features, LADA shares some of the features of both T1DM and T2DM, though it is distinct from the two. For example, complications due to LADA may arise more quickly than in T2DM but more slowly than in T1DM. Patients with LADA may have a lower risk of macrovascular complications, such as coronary heart disease, stroke, and peripheral artery disease in comparison to patients with other types of DM [308]. Patients with T2DM and LADA also have similar cardiovascular outcomes. In addition, in contrast to T1DM, LADA may be involved in the development of other autoimmune diseases and is characterized by a higher prevalence of autoimmune diseases such as autoimmune thyroid and gastrointestinal diseases [301]. Based on unique characteristics of LADA, it is clear that further work is needed to develop targeted and efficient management strategies for its treatment.

##### Management and Treatment

Currently, management and treatment strategies are associated with preserving islet β-cell function and achieving optimal blood glucose levels. However, due to the unique nature of LADA, any therapeutic strategy needs to incorporate elements from both T1DM and T2DM. Nevertheless, a key role in LADA management is played by insulin and hypoglycemic drug therapy, such as sulfonylureas, dipeptidyl peptidase IV inhibitors, and insulin sensitizers.

#### 2.2.2. Maturity-Onset Diabetes of the Young

While maturity-onset diabetes of the young (MODY) was first described in 1975 by Tattersall and Fajans [333], the term *MODY* was coined by Fajans in 1964 [334]. MODY, also known as *monogenic diabetes*, is distinct from T1DM and T2DM [335,336]. The estimated prevalence of MODY in European populations is approximately 1–5 per 10,000 individuals, corresponding to 1/10,000 or adults and 1/23,000 of children [337]. Even so, MODY is known to account for 1–5% of all cases of DM in Europeans [337,338]. It nevertheless shares some features, which may result in misdiagnosis [336]: like T1DM, it has a young age of onset, and like early-onset T2DM, it has a low risk of ketosis and can be caused by overweight or obesity [339]. As a result, almost 80% of MODY cases are misclassified as T1DM or T2DM [340].

However, the prevalence of MODY in Africa, the Middle East, and South America remains unclear [341].

Despite its rarity, MODY is the most common form of monogenic DM [342]. The condition is autosomal dominant, with transmission spanning at least three generations (i.e., grandparents, parents, and children) [343]. The diagnostic criteria comprise early onset of DM (<25 year of age), history of DM in two (not three as above) consecutive generations, DM in least two, or ideally three, family members (parents, brothers, sisters, and children), the absence of pancreatic β-cell autoantibodies, non-insulin dependence, lack of obesity, no history of DKA, lack of IR, and preserved β-cell function, i.e., lack of insulin treatment or serum C-peptide levels >200 pmol/L after three years of insulin therapy [336,341,344,345,346,347,348].

##### Subtypes of MODY

As mentioned above, MODY is a monogenic type of DM caused by mutations in single genes [349] involved in the development, function, and regulation of β-cells; they are also associated with glucose sensing and secretion of insulin [350,351]. Currently, fourteen distinct MODY subtypes have been identified based on mutations in single genes. The prevalence of these mutations varies according to inter alia geographical location and ethnicity, clinical features, and the severity of diabetes [336,338,339,349,352,353,354]. Some rare MODY subtypes may demonstrate clinical and genetic heterogeneity compared to other types; for example, one rare type may be associated with obesity and DKA, which are absent in the clinical characteristics of frequent MODY subtypes [355]. Recent research based on modern DNA sequencing techniques has identified 15 novel genes that may potentially be associated with MODY [341]. Among the 14 subtypes, mutations in genes with subtypes MODY1, MODY2, and MODY3 are found in more than 95% of MODY cases [349]. It is also suggested that not all 14 subtypes are true MODYs [355].

1. MODY1 (*HNF4A*-MODY) is associated with mutations in the hepatocyte nuclear factor 4α (*HNF-4α*) gene, located on chromosome 20q13.12 [356,357]. To date, over 400 mutations, such as missense, frameshift, splice site, nonsense, indel, deletion, and insertion, have been described [358,359,360]. For example, over 100 substitution and frameshift mutations have been detected [345]. Hepatocyte nuclear factor 4α protein is a β-cell transcription factor belonging to the receptor family of ligand-activated transcription factors [361]. It is responsible for the regulation of hepatic and β-cell expression, and it is involved in inter alia hepatocyte differentiation, the development of the gastrointestinal tract, and lipid and glucose metabolism [345,362].

The prevalence of MODY1 is the third most common form of MODY overall, representing 5–10% of among all forms of MODY [358,363]. It is characterized by progressive β-cell dysfunction, neonatal transient hyperglycemia, macrosomia/hepatomegaly history, progressive insulin deficiency, and the onset of DM in late adolescence or by the age of 25 years. Patients with MODY1 also exhibit dyslipidemia, *viz*. elevated LDL, low HDL, and low triglyceride levels [343,345,349,364]. While the patients are sensitive to low-dose sulfonylureas, they may be also treated with insulin [349].

2. MODY2 (*GCK*-MODY) is associated with mutations in the glucokinase (*GCK*) gene, localized on chromosome 7p15-p13 [356,357]. Glucokinase, also known as hexokinase IV or D, controls the secretion of insulin from pancreatic β-cells [365]. In the hypothalamic, pituitary, and gut endocrine cells, glucokinase can play a role as a glucose sensor [365]. To date, over 600 mutations, such as missense nonsense, frameshift, and splice site mutations, have been described [366]. MODY2 is the most common form of MODY in childhood and the second most common in adulthood [358]. Its reported prevalence is approximately 22% across all age groups [358] and is estimated to constitute 30 to 70% of all incidences of MODY [343]. It is also diagnosed in 88–95% of all pediatric MODY cases [367,368]. Unlike other subtypes, MODY2 is characterized by mild fasting non-progressive hyperglycemia, often asymptomatic, due to impaired insulin sensing by pancreatic β-cells [369]. In patients with activating mutations in the *GCK* gene, hypoglycemia is observed, secondary to constitutive hyperinsulinism [358]. Heterozygous inactivating mutations are indicative of MODY2 [370], while compound heterozygous or homozygous mutations are diagnosed as permanent neonatal diabetes (PNDM) [371,372]. In the case of neonatal diabetes, MODY2 is inherited in an autosomal recessive manner [362].

As patients with MODY2 are frequently asymptomatic, they are diagnosed when blood glucose is measured incidentally, for example, during pregnancy [373]. As such patients exhibit defects in glucose sensing, glucose homeostasis is maintained at a higher point; this defect causes mild and asymptomatic fasting hyperglycemia, present from birth, as well as minor postprandial glucose excursion [337]. MODY2 is generally not a progressive disease. The majority of patients do not require pharmacotherapy. During pregnancy, to treat maternal hyperglycemia, the mother should be treated with insulin to reduce the risk of diabetic fetopathy such as fetal macrosomia in the child. Serial ultrasound biometry should be performed to confirm whether the fetus has inherited the maternal glucokinase mutation. If this is the case, the fetal pancreas synthesizes larger amounts of insulin, resulting in the development of macrosomia [344]. If there is no evidence for accelerated growth, this may suggest that the fetus has inherited the GCK mutation. In this case, the maternal hyperglycemia is not treated [374].

Finally, the long-term fasting hyperglycemia observed in MODY2 is rarely associated with micro- and macrovascular diabetic complications [344]. Typically, hyperglycemia due to *GCK* mutation is diagnosed at a median age of 24 years [375]. Usually, pharmacotherapy is not initiated.

3. MODY3 (*HNF1A*-MODY) is due to mutations in hepatocyte nuclear factor 1α (*HNF1α*), localized on chromosome 12q24.31 [349,357]. The *HNF1α* gene is expressed in the liver, pancreas, kidneys, and intestine [357]. Hepatocyte nuclear factor-1α (HNF1α) protein belongs to the nuclear receptor family of transcription factors and controls the expression of insulin and insulin receptor genes in mature β-cells [376], GLUT1, and GLUT, as well as in SGLT2 [377,378]. During embryonic development, it regulates intestinal cell growth and cell differentiation. In the intestine and kidneys, HNF1α is involved in the transcriptional regulation of bile acid transporters [358], while in the liver, it is involved in gluconeogenesis and apolipoprotein synthesis. Evidence indicates that HNF1α upregulates the anti-apoptotic gene BCL2 Like1 (*BCL2L1*) [379]. This subtype is the most common MODY type, representing 30–70% of all MODY cases [362,380], and the second most common subtype of MODY diagnosed in children [358].

The observed prevalence of MODY3 among all MODY cases ranges from 15% in Polish populations to 52% among Scandinavian and British populations [358,381]. Mutations in the *HNF1α* gene, associated with MODY3, are most common in Europe, North America, and some countries in Asia [344]. Studies of approximately 1200 families have identified over 400 different mutations [361]. In this gene, the highest numbers of mutations have been observed in exon 2 and exon 4 and the lowest in exon 5 and exon 10 [358,361]. Several types of mutations have been detected, such as missense, nonsense, splice mutation, in-frame amino acid deletion, insertion, duplication, as well as partial and whole gene deletion [382].

Patients with MODY3 exhibit transient neonatal hyperinsulinemic hypoglycemia and progressive hyperglycemia throughout childhood, caused by glucosuria associated with a progressive reduction in glucose-stimulated insulin secretion. In most patients with MODY3, the onset of DM is diagnosed by 25 years of age [349,363,381,383]; data indicate that 63% of patients develop DM before 25 years, 79% before 35 years, and 96% before 55 years [335,382,384]. Patients with MODY3 often develop micro- and macrovascular complications, with one of the most prevalent being retinopathy [385,386]. The incidence of micro- and macrovascular complications is similar to T1DM and T2DM [387], and 66% of deaths in MODY3 carriers have been attributed to cardiovascular disease or cerebral vascular events [386]. The mechanism of cardiovascular disease in MODY is unclear; however, several hypotheses have bene proposed [376]. Patients with MODY3 are initially treated via diet or low-dose sulfonylureas, although insulin may be required at later stages of disease or during pregnancy [335,388].

4. MODY4 (*IPF1/PDX1*-MODY) is caused by mutations in pancreatic duodenal homeobox-1 (*PDX1*), also known as insulin promoter factor 1 (*IPF1*), located on chromosome 13q.1. The PDX1 protein acts as a transcription factor [335,345] that regulates the expression of genes that code for insulin, glucagon, GLUT2, glucokinase, somatostatin, and islet amyloid polypeptide [389,390]. The PDX1 transcription factor is also involved in the regulation of pancreatic development, β-cell maturation, and duodenal development [391]. It also serves as a master switch for both hormonal and enzymatic function of the pancreas [392].

MODY4 is a very rare subtype of MODY. However, over 150 mutations have been noted in the *PDX1* gene, including missense, nonsense, insertions, and deletions [337,345]. Heterozygous mutations in the *PDX1* gene cause β-cell dysfunction and defective insulin secretion. Homozygous mutations cause PNDM and exocrine pancreatic insufficiency [393,394,395]. A single nucleotide deletion within codon 63 (Pro63fsdelC) of the human *PDX1* gene causes pancreatic agenesis [396]. A single nucleotide substitution change at codon 33 (CCT to ACT) alters the amino acid sequence (P33T) in the IPF1 transactivation domain, resulting in complicated pregnancies; the change may also be involved in development of gestational diabetes [397]. Heterozygous mutations in *PDX1* gene may be associated with various complications, such as dorsal pancreatic agenesis, exocrine pancreas deficiency, or diabetic retinopathy [398].

Management of MODY4 includes metformin, dipeptidyl peptidase-4 inhibitor (DPP-4), oral anti-diabetes drugs (OADs), insulin, and diet [349,357].

5. MODY5 (*HNF1β*-MODY) is caused by mutations in the hepatocyte nuclear factor 1 beta (*HNF-1β*) gene, located on chromosome 17q12 [364]. The HNF1β protein, also known as transcription factor 2 (TCF2), belongs to the homeodomain-containing superfamily. It is expressed in a number of organs, including the liver, intestine, pancreas, genitourinary tract, and lungs, where it regulates their organogenesis [399].

MODY5 is the fourth most common form of MODY overall. Its prevalence is estimated to range from 2 to 6% of all forms of MODY [400] to 5 to 10% [399]. Approximately 50% of cases of MODY5 are caused by mutations within the *HNF1β* gene, with the remainder being associated with heterozygous whole *HNF1β* gene deletion [399,401]. To date, over 50 different *HNF1β* gene mutations have been described, such as missense, frameshift, splice site, nonsense, deletions, and insertions [337]. Heterozygous mutations in *HNF1β* gene decrease its transcriptional activity and decrease GLUT2 gene expression. Therefore, it has been suggested that the impaired insulin secretion associated with MODY5 is due to reduced GLUT2 expression in pancreatic β-cells rather than decreased insulin expression [402]. These mutations also cause β-cell dysfunction, hepatic IR, and congenital malformations often termed renal cysts and diabetes (RCAD) syndrome [403,404]. The renal malformations include renal cysts, renal dysplasia, renal hypoplasia, renal agenesis, genitourinary anomalies, tubulointerstitial nephropathy, horseshoe kidney, hypoplastic glomerular kidney disease, and urinary tract malformation [342,344,349,405]. Abnormalities in renal morphology can be detected from the 17th week of gestation [406].

Women with MODY5 may present structural defects of the reproductive system, such as vaginal aplasia, rudimentary uterus, and septate uterus. Male patients may present with epididymal cysts, seminal vesicle cysts, and hypospadias [349,407]. MODY5 can also be associated with a reduction in infant birth weight (900 g) [408]. In addition, 17q12 deletion syndrome is associated with whole *HNF1β* gene deletion, which can also cause neurodevelopmental abnormalities, such as mental retardation, autism spectrum disorders, schizophrenia, anxiety, and bipolar disorder [345]. In addition, many patients with MODY5 demonstrate complications of diabetes, cardiovascular risk factors, diabetic retinopathy, and neuropathy. About 50% of patients exhibit impaired renal function that may require dialysis or kidney transplantation [409].

To prevent micro- and macrovascular complications, patients with MODY5 require early therapy. In this case, the administration of sulfonylureas is inefficient and is generally not recommended [343,410], although some studies have reported effective treatment [357]. Early insulin administration is necessary [410].

6. MODY6 (*NEUROD1*-MODY) is associated with mutations in the transcription factor neurogenic differentiation 1 (*NEUROD1*) gene located on chromosome 2q32. This gene encodes for the neurogenic differentiation (NEUROD1) protein, also called β2 [411]. NEUROD1 is a type II transcription factor in the NeuroD family of basic helix-loop-helix (bHLH) factors. It is expressed in pancreatic islet β-cells, the intestine, and a subset of neurons in the central and peripheral nervous system. NEUROD1 regulates pancreatic islet morphogenesis and neuronal development [412]. It binds and activates the promoter of the sulfonylurea receptor 1 (SUR1), GCK, paired box 6 (PAX6), also known as anirida type II protein (AN2), and the glucose-6-phosphatase (G6Pase) catalytic subunit-related protein [335]. Some of these components are involved in the maintenance of normal glucose homeostasis [413,414].

MODY6 is a very rare subtype of MODY, accounting for fewer than 1% of all MODY patients. It presents with various phenotypes due to variable degrees of penetrance of the gene mutation [345]. Currently, 16 missense or truncated mutations of the *NEUROD1* gene have been genetically characterized [357]. Heterozygous mutations can cause childhood- or adult-onset diabetes due to β-cell dysfunction, while homozygous mutations can cause neonatal diabetes, neurological abnormalities, and learning difficulties [412,415,416]. Patients with MODY6 may exhibit neuropathy, low C-peptide, high HbA1c, DKA, and renal failures [414,417]. Hyperglycemia may be accompanied by obesity and neurological manifestations, such as speech delay, mental retardation, hippocampal hypoplasia, minor cerebellar dysfunction, and cerebral atrophy [342,417]. Some patients may report hearing loss and convulsions, while many others exhibit microvascular complications, such as retinopathy, neuropathy, and nephropathy as an effect of glucotoxicity [417].

A common treatment option is insulin therapy; however, patients with DM with incomplete penetrance require oral anti-diabetes (OADs) and diet management [418].

7. MODY7 (*KLF11*-MODY) occurs in response to mutations in the Krüppel-like factor (*KLF11*) gene, located on chromosome 2q25.1 The gene encodes the KLF11 protein, which is a zinc-finger nuclear transcription factor. KLF11 is expressed ubiquitously in human tissues [419]. In pancreatic islet cells, it controls β-cell function, thus acting as a glucose-inducible regulator of insulin in pancreatic and duodenal homeobox1 (PDX1) gene expression [419,420]. It is involved in terminal neuronal differentiation and neuron migration in the cerebellum, hippocampus, inner ear, and retina [345]; it also upregulates the expression of free radical scavengers, such as catalase and superoxide dismutase (SOD) [349]. KLF11 also regulates certain genes involved in the metabolism of cholesterol, prostaglandins, neurotransmitters, fat, and sugar [421,422,423,424].

MODY7 is an extremely rare disorder [425,426,427] associated with four missense mutations, *viz*. R29Q, Q62R, T220M, and A347S [419,428], and MODY7 can manifest as different phenotypes. This observation suggests that the mutated gene in MODY7 has variable penetration [425,426]. For example, in one study, the members of one family were found to share the same mutation but exhibit completely different phenotypes: the proband presented polydipsia, polyuria, and weight loss, while other members of family were silent carriers or asymptomatic with impaired glucose tolerance [429]. Some of these patients were lean, whereas others had obesity [426,427,429]. Some mutations in the KLF11 gene cause very-early-onset DM type 1b [430], while other cause later-onset DM [431,432].

Clinically, patients with MODY7 can be characterized by pancreatic malignancy [357]. MODY7 is managed with insulin or OADs [335].

8. MODY8 (*CEL*-MODY) is caused by heterozygous mutations in the carboxyl ester lipase (*CEL*) gene located on chromosome 9q34.13 [358]. It is expressed primarily in the acinar tissues of the pancreas and secondarily in lactating mammary glands [433,434]. The *CEL* gene contains a variable number of tandem repeats (VNTR) region that encodes a mucin-like protein tail. The gene encodes carboxyl ester lipase (CEL), also known as bile salt-dependent lipase or bile salt-stimulated lipase [357]. CEL hydrolyzes fat-soluble vitamins, dietary fat, and cholesterol esters in the duodenum and is involved in their absorption [435]. The *CEL* gene is known to harbor missense, frameshift, deletion, and insertion mutations [337].

The primary results of *CEL* gene mutation include early pancreatic atrophy, subsequent exocrine insufficiency caused by exocrine pancreatic tissue and not the islets, pancreatic lipomatosis, pancreatic cysts late in life, and endocrine dysfunction. These pathologies, caused by CEL enzyme misfolding and cytotoxic aggregation, are caused by single-base-pair deletions in the VNTR repeat [436,437,438,439]. Some patients have pancreatitis [440]. MODY8 manifests as DM in adulthood [440]. Some studies also note the presence of microvascular complications, such as non-proliferative diabetic retinopathy and peripheral neuropathy [342].

MODY8 is a rare MODY subtype. Its management includes dietary, OADs, and insulin interventions [357].

9. MODY9 (*PAX4*-MODY) is caused by nutation in the paired homeobox4 (*PAX4*) gene, located on chromosome 7q32.1. This gene encodes the nuclear transcription factor paired box4 (PAX4) protein, required for insulin production and pancreatic β-cell differentiation, development, and maintenance. The transcription factor also regulates β-cell survival [349,357]. During the early stage of embryonic development, PAX4 is expressed in endocrine promoter cells and, later, in pancreatic β-cells [441]. The gene has been found to suffer various missense, deletion, and splice site mutations.

While the mean age of diagnosis is typically the third decade of life, it has also been diagnosed at 19 months old [358,442]. Patients with MODY9 exhibit polydipsia, polyphagia, polyuria, and sometimes DKA [349,358,442]. Basal insulin secretion is also absent, resulting in an elevated risk of ketosis-prone diabetes [443]. Diabetic complications such as retinopathy and nephropathy have been also described, and in some cases, it can have a fatal course due to end-stage renal failure [342]. Treatment of MODY9 is effectively management by diet, OADs, and insulin [335].

10. MODY10 (*INS*-MODY) is caused by mutations in the insulin (*INS*) gene, located on chromosome 11p15.5. The *INS* gene encodes preproinsulin, which is cleaved to proinsulin and then to insulin. Autoimmune destruction of β-cells causes autoimmune T1DM, while various genetic and environmental factors cause IR, insulinopenia, and T2DM. Mutations in the *INS* gene cause misfolding and intracellular accumulation of proinsulin, defective insulin synthesis, and defects in nuclear factor-kappa-light-chain-enhancer of activated β-cells (NF-κβ) [444,445]. Prolonged endoplasmic reticulum stress activates the terminal unfolded protein response, resulting in the induction of β-cell apoptosis [337].

Different pathologies can be detected depending on the mutation. For example, heterozygous missense mutations in the *INS* gene can cause protein misfolding, protein aggregation, endoplasmic reticulum stress, elevated β-cell apoptosis, decreased β-cell mass, and gradually the loss of insulin secretion, whereas homozygous mutations cause a loss of function and decreased insulin biosynthesis due to gene deletion. Both heterozygous and homozygous mutations can result in at least an 80% decrease in insulin production [357].

Mutations in the *INS* gene are a frequent cause of isolated PNDM but are less likely to result in DM diagnosed in children or adulthood. This dependence is more commonly observed when the diagnosis is made before one year of age [446]. Some families have reported complications, such as mild proliferative diabetic retinopathy, neuropathy, and polycystic ovarian syndrome (PCOS) [342]. Clinically, patients with MODY10 are characterized by neonatal hypoglycemia [357].

This MODY subtype is effectively managed by diet, OADs, or insulin [335]. Other therapies including islet transplantation and stem cell-derived β-cell replacement have been proposed [364].

11. MODY11 (*BLK*-MODY) occurs in response to mutations in the specific tyrosine-protein kinase B lymphocyte kinase (*BLK*) gene, located on human chromosome 8p23.1 [364]. The *BLK* gene belongs to the SRC family of proto-oncogenes. Its preferential sites of expression include B lymphocytes and β-cells [357]. The BLK protein plays a role in β-cell signaling and β-cell development. It also upregulates the transcription factors PDX1 and NKX6.1, which are involved in the synthesis and secretion of insulin [447].

As MODY11 has incomplete penetrance, not all carriers have diagnosed DM [335]. Similarly, the p.A71T mutation also demonstrates incomplete penetrance; it is a loss-of-function mutation that can negate the enhancing effect of BLK on insulin content and secretion from pancreatic β-cells [448]. While the mutation may act as a diabetogenic factor in patients with obesity and overweight, it has demonstrated a weak correlation with DM [448]. Therefore, it is suggested that environmental and genetic modifiers may play an important role in MODY11. This relationship is particularly apparent in patients with obesity and overweight, which are the most important factors associated with hyperglycemia [448]. No complications associated with MODY11 have been reported [342]. MODY11 is a very rare MODY subtype [343]. Some patients may be treated with diet or OADs, but most patients require insulin therapy [447].

12. MODY12 (*ABCC8*-MODY) is caused by mutations in the ATP-binding cassette transporter subfamily C member 8 (*ABCC8*) gene, located on chromosome 11p15. It is highly expressed in the pancreas, where it encodes sulfonylurea receptor 1 (SUR1), a subunit of the ATP-sensitive potassium (K-ATP) channel detected in pancreatic β-cell membranes. The K-ATP channel regulates glucose-stimulated insulin secretion from pancreatic β-cells. The *ABCC8* gene has been found to harbor over 125 mutations, of which the most common were missense types [357]. MODY12 can cause congenital hypoglycemia, hyperinsulinism, transient neonatal diabetes (TNDM) or PNDM, or adulthood-onset DM [449,450,451]. For neonatal diabetes, PNDM is associated with homozygous mutations and TNDM with heterozygous mutations [344]. Some gain-of-function and loss-of-function mutations in the *ABCC8* gene can cause transient neonatal hyperinsulinism with DM later in life [452,453]. The inheritance of neonatal diabetes can be dominant, often de novo, or recessive [449,454,455].

The clinical manifestations vary between patients. In some, weight loss, polyphagia, polyuria, ketosis, and low serum insulin levels can be observed at a young age. Others may present with impaired glucose tolerance and mild hyperglycemia. MODY12 can be associated with different forms of microvascular complications [456]. Patients with MODY12 are clinically characterized by renal diabetes [357]. MODY12 is rare MODY subtype, with an estimated prevalence <1% of MODY [449]. MODY12 is effectively treated with sulfonylureas and insulin [357].

13. MODY13 (*KCNJ11*-MODY) is caused by mutations in the potassium inwardly rectifying channel subfamily J member 11 (*KCNJ11*) gene, mapped to chromosome 11p15.1 [357]. *KCNJ11* gene is highly expressed in the pancreas, where it encodes for pancreatic β-cell inwardly rectifier (BIR), the pore-forming KIR6.2 subunit of the ATP-sensitive potassium (K-ATP) channel, which plays an important role in the secretion of insulin. Gain-of-function and loss-of-function missense mutations in the *KCNJ11* gene inactivate the channel following disrupted subunit interaction [349,357]. Mutations in the *KCNJ11* gene are associated with PNDM [335] and with TNDM [457]. Loss-of-function mutations cause congenital hyperinsulinism [451].

Patients with MODY13 may experience convulsions caused by extreme hypoglycemia, neurological abnormalities, such as developmental delays, autism, ADHD, and seizures [357], and the development of DM due to impaired insulin secretion [448]. Many patients are characterized by renal diabetes, hyperinsulinemic hypoglycemia, and neonatal DM [357].

MODY13 is inherited as an autosomal dominant disease, with neonatal diabetes as dominant or often de novo [448,458]. MODY13 is a rare subtype constituting <1% of MODY cases [380]. Patients with MODY13 are effectively treated with sulfonylureas [357].

14. MODY14 (*APPL1*-MODY) is caused by mutations in the adaptive protein phosphotyrosine, which interacts with the PH domain and leucine zipper-contacting *APPL1* gene, located on chromosome 3p14.3 [349]. This gene is highly expressed in skeletal muscle, the pancreas, liver, endothelium, and adipose tissue [337]. The APPL1 protein binds to RAC-beta serine/threonine-protein kinase or AKT serine/threonine kinase 2 (AKT2). The APPL1 protein is involved in the intracellular insulin signaling pathway and interacts with AKT2, IRS-1, IRS-2, and the insulin receptor (INSR) [459,460]. It also controls insulin-stimulated glucose uptake in skeletal muscle and adipose tissue [337], as well as cell proliferation, interactions between adiponectin and the insulin signaling pathway, and the inhibitory effect of insulin on hepatic gluconeogenesis [461]. Mutations in the APPL1 protein result in premature apoptosis of highly expressed, tissues, and protein overexpression is associated with dysmorphic phenotypes and developmental delay [461]. Heterozygous missense loss-of-function mutations in this gene impair insulin secretion in response to glucose stimulation and decreased β-cell survival [461,462]. MODY14 mostly develops after 40 years of age, [462] but has been found to occur in patients from 20 to 50 years of age [461,462]. MODY14 is a very rare subtype. Patients are characterized by Wolfram syndrome [357]. Possible treatments include diet, OADs, and insulin administration [335].

#### 2.2.3. Neonatal Diabetes Mellitus

The second type of monogenic DM is neonatal diabetes mellitus (NDM), also known as *congenital diabetes* or *diabetes of infancy*. The cause is believed to be a monogenic defect. In newborns, it is important to distinguish NDM from other causes of hyperglycemia, such as infection, stress, and inadequate pancreatic β-cell production of insulin in preterm infants [463]. NDM is defined as insulin-requiring hyperglycemia or as the onset of diabetes before the age of six months [464].

##### Prevalence of NDM

Diabetes mellitus due to a single gene mutation, i.e., *monogenic diabetes*, is a rare form of DM. It is estimated to account for 1–5% of all DM cases; however, its prevalence depends on the studied population [465,466].

While NDM constitutes 50% of monogenic diabetes cases, it is a rare disease, with an estimated incidence of 1 in 300,000 to 400,000 newborns; however, a higher incidence has been identified in some European countries (i.e., 1 in 89,000 newborns) [467,468]. Other studies report an incidence of 1 in 500,000 live births [469] or 1 in 90,000 to 160,000 live births globally [470]. A particularly high incidence (1 in 20,000) has been noted in northwest Saudi Arabia [471]. The incidence has been found to be 1 in 45,000 in Oman and to range from 1 in 400,000 to 1 in 900,000 live births in the UK, Germany, and Slovakia [472].

##### Clinical Features and Diagnosis of NDM

While NDM usually arises within the first six months of life, it may also occur between six and twelve months of life [471,473]. It has been proposed that the occurrence of NDM after the sixth month may be due to autoimmune diabetes rather than the monogenic form [474]. NDM can be classified into transient neonatal diabetes (TNDM), which remits early and may recur in later life [466,475], and PNDM, which requires lifelong therapy [476].

Neonates with NDM commonly exhibit intrauterine growth restriction (IUGR) and low birth weight due to disturbances in the synthesis and secretion of insulin, which plays an important role in normal embryonic growth [477]. Patients may also experience hyperglycemia, muscle fatigue, biasness in growth and development, low or undetectable C-peptide levels, and difficulty in cognition in patients with ketoacidosis [463]. Some manifestations may be insidious, such as failure to thrive, and some characteristic symptoms of DM, such as polyuria and polydipsia, are often difficult to recognize in infants [463,473]. In some infants with NDM, clinical features of diabetes may suggest a sepsis-like illness or DKA [468,478,479].

A diagnosis of NDM should be considered if severe hyperglycemia in the neonate persists beyond a week or if extremely high glucose levels (>1000 mg/dL, >55.5 mmol/L) are detected [463,480].

##### Association Between Genetic Factors and NDM

NDM is caused by single-gene mutations affecting the secretion of insulin; these have been recorded in more than 40 genes. These mutations affect the development and functions of pancreatic β-cells, including the synthesis and secretion of insulin [470,480,481]. The evidence suggests that the development of NDM may involve 49 genes [472], some of which are indirectly involved in the condition, and over 20 are known to be crucial for the development of pancreatic β-cells [482,483].

The most common mutations associated with NDM are detected in two genes that encode subunits of the pancreatic β-cell K_ATP_ channel, *viz*. the potassium inwardly rectifying channel subfamily J member 11 (*KCNJ11*) gene and ATP-binding cassette transporter subfamily C member 8 (*ABCC8*) gene, and mutations in the insulin gene (*INS*). Patients with mutations in the K_ATP_ channel subunit genes may develop DEND syndrome, characterized by the triad of developmental delay, epilepsy, and NDM [484,485].

The genetic causes of NDM with normal pancreas morphology include various abnormalities of the 6q24 locus [471], among others [478,486]. The *KCNJ11* gene, located on chromosome 11p15.1, encodes Kir6.2, the inner subunit of the ATP-sensitive potassium (K_ATP_) channel of the β-cell membrane. This gene is inherited in an autosomal dominant pattern. In many cases, genetic change occurs as a de novo mutation [486]. The *ABCC8* gene, also mapped to chromosome 11p15.1, encodes sulfonylurea receptor 1, the outer subunit of K_ATP_ channel [487]. It is inherited in an autosomal dominant or recessive manner or as a new mutation.

The K_ATP_ channel plays a role in the stimulation of insulin secretion by the pancreatic β-cell in response to glucose. At low blood glucose levels (e.g., fasting), K_ATP_ channels are activated and are open; this activity maintains a hyperpolarized resting membrane. Following an increase in blood glucose level (e.g., post-prandial), glucose is taken up into pancreatic β-cells, where it enters the glycolysis pathway. The intracellular ATP concentration then increases, causing intracellular potassium accumulation and membrane depolarization. Membrane depolarization activates voltage-dependent calcium channels, increasing the inflow of Ca^2+^ into the β-cell, which facilitates the exocytosis of secretory vesicles and the release of insulin into the bloodstream [471,488]. Mutations in *KCNJ11* or *ABCC8* cause the K_ATP_ channel to remain permanently open, preventing it from controlling the membrane potential in response to rising glucose levels; as a result, insulin secretion is impaired [471,488].

NDM can also result from abnormalities in the 6q24 locus such as paternal uniparental disomy of the 6q24 locus (pUPD6), partial duplication of paternal 6q24, and relaxation of the maternal 6q24 impaired locus [489]. The locus harbors a CpG island whose methylation depends on parental origin: normally, a CpG island is unmethylated on the paternal allele and methylated on the maternal allele. This methylation status causes the expression of the paternal allele, and the maternal allele is inactive [468,471].

In addition, NDM can result from the overexpression of imprinted genes, such as pleomorphic adenoma gene-like 1 (*PLAGL1*) and the hydatiform mole-associated imprinted transcript (*HYMAI*) [490,491]. *PLAGL1* is a transcription factor whose overexpression in pancreatic β-cells increases the expression of p21 and whose interaction with the tumor suppressor p53 reduces cell proliferation and enhances apoptosis [492,493]. The *HYMAI* gene encodes a non-coding RNA, whose function remains unclear [478,494]. Animal studies indicate that *PLAGL1* and *HYMAI* overexpression impair the excretion of insulin and the development of pancreatic endocrine development resembling TNDM in humans [478,494].

The occurrence of NDM due to abnormalities at the 6q24 locus occurs before one month of age in 93% of cases and before three months in 100%. In 97% of patients with 6q24 locus abnormalities, remission is observed before one year of age. These patients may have developmental defects, such as macroglossia, umbilical hernia, cardiac renal and urinary malformations, non-autoimmune anemia, hypothyroidism, and neurological disorders [495].

The third most common cause of NDM and the second of PNDM is the presence of mutations in the insulin gene, located on chromosome 11p15.5. In the case of TNDM, these mutations are rare [471]. The majority are heterozygous mutationsl which affect the structure of preproinsulin and are inherited in an autosomal dominant manner [496]. To date, more than 70 mutations have been described [480]. Structural abnormalities in preproinsulin cause it to be degraded in the endoplasmic reticulum, resulting in severe endoplasmic reticulum stress and β-cell death [497,498].

Patients with NDM demonstrate dysfunctional insulin secretion, which may occur via heterozygous gain-of-function mutations or homozygous loss-of-function mutations in the *INS* gene [481]. In more than 80% of patients with dominant pathogenic variants, proinsulin is misfolded and accumulates within endoplasmic reticulum [471]. More than 80% of heterozygous mutations in the *INS* gene are de novo, while the remainder are inherited in an autosomal dominant manner [446]. Recessive loss-of-function mutations, homozygous or compound heterozygous, markedly reduce or suppress the biosynthesis of insulin.

Preproinsulin synthesis may be reduced by different mechanisms [499,500,501]. Patients with NDM due to an *INS* gene mutation frequently exhibit IUGR compared to those caused by other genetic factors. Most patients are diagnosed during the first six postnatal months [495]. Lack of insulin biosynthesis caused by the prenatal effects of recessive mutations result in severe growth restrictions, whose effects are more severe and occur earlier, i.e., within the first postnatal days [487,495,502]. In these patients, no extra-pancreatic features are present [474]. Patients with NDM due to mutation in the insulin gene may appear clinically similar to patients with early-onset T1DM. While these patients have not been found to demonstrate neurodevelopmental delay, some patients may exhibit inter alia depression or anxiety, which are common in all forms of DM [473].

Glucokinase, encoded by the glucokinase gene (*GCK*), located on chromosome 7p15.3-p15.1, plays a role as a glucose sensor in pancreatic β-cells, regulating the amount of insulin secreted. The protein catalyzes the first step of glucose metabolism and is involved in the maintenance of normoglycemia [495]. Since glucokinase regulates insulin secretion, mutations in the *GCK* gene are associated with hyperglycemia and hypoglycemia. To date, 700 mutations have been described. Homozygous or compound heterozygous loss-of-function mutations cause the complete loss of *GCK* activity, resulting in PNDM; however, the occurrence of NDM due to *GCK* mutations is rare, accounting for only 2–3% of PNDM cases [480]. Mutations in *GCK* commonly result in IURG and hyperglycemia presenting during the first days of life. No associated extra-pancreatic malformations are present [495].

Mutations in the *ABCC8* and *KCNJ11* genes play a fundamental role in the development of NDM, and they are present in some patients. While causative mutations have been noted in other genes, they are rarely the cause of NDM [468,471,473,478,480,481,486,503].

##### Characteristics of Types of NDM

Neonatal diabetes mellitus has been classified into two subtypes: TNDM and PNDM.

- Transient Neonatal Diabetes Mellitus

Transient neonatal diabetes mellitus (TNDM) is a rare insulin-dependent DM with an incidence of approximately 1 in 90,000 to 160,000 live births [472], contributing to 50 to 60% of NDM [504]. In children with TNDM, hyperglycemia occurs in infancy, i.e., before 13–18 weeks of age, and usually resolves within 12 weeks; most patients recover within one year, and its occurrence may be in later adolescence and early adulthood [463,486,505]. In adolescents or adults, TNDM may convert to a permanent DM state [504].

Around 70% of cases are caused by mutations in chromosome 6q24 and 25% by mutations in the *ABCC8* and *KCNJ11* genes [506,507]. The chromosome region 6q24 contains two major genes associated with TNDM: *HYMAI* and *ZAC1*. These genes are generally linked with TNDM. ZAC is a C2H2 zinc-finger transcription factor. It plays multiple roles, such as being a coactivator of apoptotic protease activating factor 1 (Apaf1) transcription, and together with p53, it regulates the histone acetyltransferase activity of p300 and acts as a coactivator or corepressor of various nuclear hormone receptors [508]. HYMI is an untranslated RNS whose function remains undetermined [509].

These genes may be overexpressed in response to paternal duplication, paternal uniparental disomy, and abnormal methylation of the maternal allele [509,510]. Uniparental disomy of chromosome 6 is sporadic. Therefore, the risk of paternal duplication of the 6q24 region has a 50% chance of transmitting the abnormalities and TNDM to the children. When the mutation is on the maternal side, the duplication will be passed on, and the children will not develop TNDM; however, the disease may recur instead in the next generation [486]. Patients with TNDM caused by mutations in the 6q24 region, born IURG, usually develop severe non-ketotic hyperglycemia during the first week of life [509,511]. After birth, such patients often fail to thrive, with some experiencing dehydration.

Mutations in the *KCNJ11* and *ABCC8* genes cause the K_ATP_ channels to remain open despite hyperglycemia, preventing the cell membrane from depolarizing and releasing insulin [478]. In contrast to patients with 6q24 abnormalities, those with TNDM caused by the activation of K_ATP_ channel mutations show milder IURG and are diagnosed later, suggesting that the prenatal deficiency is less severe. These patients also exhibit later remission of NDM and earlier relapse [512].

One report describes the interesting case of a one-month-old male infant with TNDM whose cause was unknown [513]. Hyperglycemia was noted in the neonatal period. The boy was born at 38 weeks of gestation, with IURG and a birth weight of 2080 g. The mother had been diagnosed with GDM that had been treated with insulin. The paternal grandfather and aunt had T2DM. The physical examination of the patient revealed some characteristic features, such as an enlarged braincase in comparison to the facial skeleton, macroglosia, umbilical hernia, and hydrocele in both testicles. This was accompanied by hyperglycemia and fluctuations in glycemic levels. Additional analyses confirmed correct acid–base homeostasis and normal blood ketone levels; no glycosuria or ketonuria or infection was observed. Analysis of *ABCC8*, *EIF2AK3*, *FOXP3*, *GCK*, *GLIS3*, *HNF1A*, *HNF1B*, *HNF4A*, *INS*, *KCNJ11*, *PDX1*, and *PTF1A* did not identify any pathogenic or potentially pathogenic variants; no pathogenic or potentially pathogenic variant was noted in the 6q24 locus. Deletion or duplication of the 6q24 region was excluded, and no aberrations were detected in the copy number variation in this region. The test results for autoantibodies typical for T1DM were also negative. The analyses and therapy are detailed in the report [513].

- Permanent Neonatal Diabetes Mellitus

PNDM is typically diagnosed at less than six months of age [472]. PNDM is less common than TNDM. It is an infrequent syndrome, and its incidence ranges from 1 in 89,000 to 1 in 400,000 [472]. In PNDM, failure of insulin secretion occurs in the late fetal or early post-natal period. In patients with PNDM, DM develops in the neonatal period and does not enter remission [504,514].

The most common cause of PNDM is heterozygous mutations in the *KCNJ11* (over 31% of cases) and *ABCC8* (over 10% of cases) genes [515]. Of these, the most common mutations occur in *KCNJ11*, and more patients have PNDM than TNDM; however, *ABCC8* mutations more frequently result in TNDM [486] and account for more than 50% of all cases of PNDM [478]. PNDM patients with mutations in the *KCNJ11* gene exhibit various symptoms including sleep disturbance, attention-deficit/hyperactivity disorder, and delays in learning. These patients may also present with DEND syndrome.

The second most common cause of PNDM after *KCNJ11/ABCC8* gene mutations is mutations in the *INS* gene, which are detected in up to 20% of infants with PNDM [446,451]. Insulin protein misfolding can cause the death of pancreatic β-cells. The median age at diagnosis is 10 weeks, and 30% of these patients present DKA [479]. PNDM patients with mutations in the *INS* gene are diagnosed later (eleven weeks) than those with *KCNJ11/ABCC8* gene mutations (eight weeks) [446]. These patients only have phenotypic features characteristic of PNDM.

##### Management and Therapy of NDM

Infants with persistent hyperglycemia due to NDM should initially be started with insulin therapy. Effective glucose control can be achieved with insulin rates ranging as low as 0.02 units/kg/hour; in the case of infants with very low birth weight, an initial dose of 0.05 units/kg/hour can be used [516]. Insulin can be administered as an intravenous infusion, intermittent subcutaneous therapy, or continuous subcutaneous insulin infusion [468,517]. Sulfonylurea therapy can be effective for treating hyperglycemia in patients with NDM caused by mutations in *KCNJ11* or *ABCC8* [478].

##### Complications of NDM

NDM can be associated with inter alia Wolcott–Rallison syndrome, IPEX syndrome, Fanconi Bickel syndrome, Rogers syndrome, Wolfram syndrome, and Rabson–Mendenhall syndrome. Some syndromic forms also exist due to mutations in *EIF23*, *GCK*, *GLIS3*, *RFX6*, *IER3IP1*, and *MNX1* [463,518].

#### 2.2.4. Steroid-Induced Diabetes Mellitus

Glucocorticoids (GCs), also known as steroids, are a class of anti-inflammatory drugs used commonly to treat a wide variety of both acute and chronic illness, such as inflammatory diseases, immunosuppression, and anti-allergic reactions. It is estimated that as many as 1–8% of all adult patients are receiving corticosteroids [519], reaching 10% among hospitalized patients in the UK [520]. Their use is associated with numerous side effects, such as osteoporosis, sarcopenia, hypertension, cataracts, and glaucoma, and prolonged treatment may result in glucocorticoid-induced hyperglycemia (GIH) and steroid-induced diabetes mellitus (SIDM) [521,522].

The term *steroid diabetes* was introduced in the 1940s to describe GIH [523]. The administration of GCs has been found to induce hyperglycemia in rats [523,524], and GIH has also been described in humans [525,526]. The effects of GIH depend on their dose and may be observed rapidly often within hours of administration [527]. Prolonged high-dose GC therapy increases the risk of GIH, and use of GC in individuals without DM is associated with a twofold higher risk of hyperglycemia [528]. The side effects of GC also depend on the type of GC. For example, hospitalized patients treated with oral IV dexamethasone or methylprednisolone were found to be at a higher risk of GIH than those receiving prednisolone or hydrocortisone. Hospitalized patients with DM exhibited higher capillary blood glucose levels than those without DM [529]. While SIDM is common in the adult population [530], it is rare in children [531]. Even so, some cases have been noted in pediatric patients, and it has been proposed that “SIDM may not be so rare in the pediatric population as once thought” [532].

##### Definition and Diagnosis of SIDM and GIH

The classical definition of SIDM specifies it as “abnormally elevated blood glucose associated with the use of glucocorticoids in patients with or without a previous history of DM” [533]. Criteria for the diagnosis of SIDM do not differ from other types of DM and include fasting blood glucose ≥7 mmol/L (≥126 mg/dL), a glucose level of ≥11.1 mmol/L (≥200 mg/dL) in OGTT, an HbA1c ≥ 6.5% (≥48 mmol/mol), or a random blood glucose ≥ 11.1 mmol/L (≥200 mg/dL) in patients with symptoms of hyperglycemia or hyperglycemic crisis [534].

##### Pathophysiology of SIDM and GIH

The therapeutic use of glucocorticoids can have several side effects acting through various mechanisms. The drugs are used to counteract the metabolic effects of insulin and cause the upregulation of the hepatic enzymes involved in gluconeogenesis. Therapy results in increased liver glucose output and reduced glucose absorption and utilization in skeletal muscle and adipose tissue due to the inhibition of insulin responses [535]. It also stimulates lipolysis in adipose tissue, increasing IR and causing the impairment of insulin secretion from pancreatic β-cells [536,537]. These disturbances can be attributed to the impairment of the intracellular insulin signaling pathway [538].

Insulin resistance in skeletal muscle, induced by GCs, can occur in response to reduced transcription of IRS-1 and increased transcription of the protein tyrosine phosphatase type 1 (PTP1B) and mitogen-activated protein kinase (MAPK) pathways [539]. By interfering with the insulin signaling pathway, GCs inhibit the translocation of GLUT4 from the intracellular compartment to the cell membrane, resulting in IR [540,541]; this has been associated with the effect of GCs on the PI3K and PKB/AKT signaling pathways [540,542,543].

Glucocorticoid use reduces insulin production by supporting GIH; this results in decreased expression of GLUT2, involved in the transport of glucose into β-cells; treatment also decreases the amount of glucokinase receptors on pancreatic β-cells [544]. The transport of glucose into β-cells triggers several processes that result in the secretion of insulin into the blood. In heathy individuals, administration for one to seven days was found to cause IR and impair glucose homeostasis [545,546]. According to one meta-analysis, GC treatment lasting more than one month was associated with a 32% risk of GIH and 19% risk of DM in non-diabetic patients [547]. In diabetic patients, starting GC treatment may exacerbate hyperglycemia [548]. In healthy individuals, both acute and two-week exposure to GCs were found to impair pancreatic β-cell function [549].

It has been proposed that the pro-apoptotic effect of GCs may play a part in β-cell failure [550]. Indeed, GC treatment was reported to inhibit insulin synthesis due to induced apoptosis in a rat model of insulinoma [551]. The association between GCs and SIDM and GIH is presented in more detail elsewhere [521,522,529,532,538,552,553].

##### Clinical Relevance

Glucocorticoid-induced GIH and SIDM are associated with increased morbidity and mortality. GIH can give rise to osmotic symptoms and can cause acute complications, such as DKA and a hyperosmolar hyperglycemic state (HHS). It is associated with poorer outcomes, such as longer or more frequent hospitalization, infection risk, and increased morbidity and mortality [552]. It can increase the risk of microvascular and macrovascular complications and increase cardiovascular risk [544].

##### Therapeutic Management

Therapeutic management should be started for fasting blood glucose levels > 140 mg/dL and post-prandial levels > 180 mg/dL [552]. Pharmacotherapy is based around insulin secretagogues, such as sulfonylureas, insulin sensitizer agents, such as metformin, and incretins and gliflozins, as well as insulin [522,538]. Herbal management of SIDM has also been proposed [554].

#### 2.2.5. Type 3c Diabetes Mellitus

Type 3c diabetes mellitus (T3cDM) comprises secondary to pancreatic diseases, which affect the pancreas exocrine compartment and/or composite (exocrine and endocrine) pancreas; hence, it is also known as pancreatogenic DM [5,6,555,556]. It is a distinct form of DM recognized by both the ADA and WHO [557]. Unlike T1DM and T2DM, T3cDM manifests as structural and functional damage to the pancreas and disruptions to the pancreatic architecture [558]. It arises from pancreatic diseases, pancreatic resection, or injury, thus damaging the exocrine and endocrine functions of the pancreas. Pancreatic insufficiency causes insulin deficiency, glycemic variability, and maldigestion [559]. Patients with T3cDM are frequently misclassified, usually as T2DM [560].

##### Epidemiology of T3cDM

Previously, T3cDM was considered a rare type of DM; however, recent data suggest that it may account for 5–10% of DM cases worldwide [561] and 5–10% of DM patients in Western countries [562]. However, the literature can be quite inconsistent, with recent studies suggesting that the global prevalence may be as high as 12% [559], while others propose a value ranging from 1 to 9% [563,564] to approximately 2% [565].

Nevertheless, the incidence of T3cDM is higher than that of T1DM [564], and the condition appears to be less prevalent in the pediatric population than among adults [566]. Older studies indicate a prevalence of about 0.5–1.5% among all cases of DM in North America [567] and 15–20% in Southeast Asia [568,569]. Unfortunately, the T3cDM account is not clearly documented globally, and accurate estimates of this type are difficult to obtain, often due to misdiagnosis [570,571].

##### Clinical Presentation and Diagnosis of T3cDM

Many patients with T3cDM have some history of pancreatitis together with abdominal pain, steatorrhea, or maldigestion with nutritional deficiencies and glucose intolerance [565]. These patients may present with maldigestion and/or abdominal pain without a prior diagnosis of chronic pancreatitis. Early in the course of endocrine insufficiency, the changes in glucose metabolism begin as asymptomatic and mild hyperglycemia; however, this can progress to brittle DM, characterized by marked glycemic lability and frequent hypoglycemia. These disturbances of glucose metabolism occur in response to the loss of insulin secretion by islet β-cells and counter-regulatory glucagon secretion from pancreatic islet α-cells [572]. Replacement doses may cause hypoglycemia [557]. Due to this impairment in its endocrine and exocrine functions, the pancreas does not provide the body with enzymes needed for appropriate digestion, resulting in diarrhea, abdominal bloating, eating issues, bloating and abdominal pain, nausea, and general fatigue [570].

A diagnosis of T3cDM presents a challenge due to its overlap with T1DM and T2DM and the lack of clear diagnostic criteria. Many patients with T3cDM demonstrate symptoms indicative of T2DM, such as polyuria, polydipsia, and weight loss; however, while T2DM is primarily associated with IR and β-cell dysfunction, T3cDM is associated with pancreatic damage, leading to a combination of insulin deficiency and exocrine insufficiency [573]. Diagnosis is also complicated by the high variability of blood glucose levels, which can sometimes be too high and sometimes too low [565]. As a result, patients are often misclassified as T2DM; for example, in a German study, only half of the cases of T3cDM were classified as T2DM [574]. Currently, there remains no universally accepted diagnostic criteria for T3cDM. The most common diagnostic approaches include the patient’s history of pancreatic diseases, computed tomography (CT), magnetic resonance imaging (MRI), and endoscopic ultrasound. These can be accompanied by biochemical tests, such as C-peptide levels to assess β-cell function and fecal elastase to confirm exocrine insufficiency [559,561]. Due to this lack of diagnostic criteria, the best-known model is the list proposed by Ewald and Bretzel [575].

Major criteria (must be present) include the following:

* Presence of exocrine pancreatic insufficiency (monoclonal fecal elastase-1 test or direct function test;
* Pathological pancreatic imaging (endoscopic ultrasound, MRI, or CT);
* Absence of T1DM autoimmune markers.

Minor criteria are as follows:

* Absent pancreatic polypeptide secretion;
* Impaired incretin secretion (e.g., GLP-1);
* No excessive IR (e.g., Homeostasis Model Assessment of Insulin Resistance (HOMA-IR);
* Impaired beta cell function (e.g., Homeostasis Model Assessment of Beta Cell (HOMA-B), C-peptide/glucose ratio);
* Low serum levels of lipid soluble vitamins (A, D, E, and K).

The above-mentioned criteria are accepted; however, there are several suggested limitations [556,575]. For example, there still is no ideal standardized method to identify IR, so the above-mentioned criteria could be overlapping with the long-standing T1DM and T2DM criteria; apart from that, peripheral insulin sensitivity is variable.

##### Pathophysiology of T3cDM

The most common causes of T3cDM include acute pancreatitis (AP), acute recurring pancreatitis (ARP), chronic pancreatitis (CP), and cystic fibrosis (CF). In addition, pancreatic resection and various cancers and genetic disorders can also play a role [557,571].

Postpancreatitis diabetes mellitus (PPDM) refers to the occurrence of pancreatogenic diabetes resulting from pancreatitis. The risk of PPDM is determined by acute inflammation of the pancreas (AP), ARP, and CP [563]. Acute inflammation of the pancreas (AP) is characterized by inter alia sudden-onset abdominal pain and more than threefold elevations in amylase and lipase levels. ARP is defined as more than one episode of AP. CP is characterized by pancreatitis with irreversible structural changes in the pancreas [563]. It is accepted that 30% of patients with AP may develop a chronic form of disease, *viz*. ARP or CP [576]. A single attack of pancreatitis may be involved in the development of T3cDM, but the risk is highest with CP and ARP [288]. A previous meta-analysis found the risk of DM to be 15% after one year following the initial episode of AP and that this increases to 23% after five years [560].

CP affects the pancreas morphologically and physiologically. It is a fibro-inflammatory condition that causes widespread replacement of fibrotic tissue associated with recurrent incidences of pancreatic inflammation. This replacement causes exocrine and endocrine pancreatic insufficiency [577]. Studies have found CP to be the primary cause of T3cDM, i.e., in 79% of cases [578]. The prevalence of T3cDM is elevated in adults with CP and increases with the duration of CP. The lifelong risk of T3cDM ranges from 46% to 83% after 20 years of CP [579,580]. Patients with CP have a 70% likelihood of developing T3cDM, increasing to 90% in patients with calcific CP [562]. The risk of T3cDM is higher in children with ARP/CP and has been diagnosed in 4–9% [581] or in 6–9% [582] of diabetic patients depending on the study. This risk is higher in hereditary pancreatitis, where lifetime T3cDM risk approaches 70% to 80% [563]. The other causes of T3cDM include pancreatic ductal adenocarcinoma (8%), hemochromatosis (7%), cystic fibrosis (4%), and pancreatic surgery (2%) [578]. This is described in more detail elsewhere [558,563].

The development of pancreatic disease is also associated with pathogenic genetic variants. For example, genetic variant c.311T>C (p.L104P), detected in the serine protease 1 (*PRSS1*) gene, coding for cationic trypsinogen, is frequently detected in families with heredity chronic pancreatitis (HCP). It plays a role in recurrent AP and as a precursor to CP [583,584]. Approximately 48% of individuals with this pathogenic variant will develop DM, including T3cDM [585].

##### Complications

Little is known about the complications associated with T3cDM. It has been suggested that patients with T3cDM have a similar risk of micro- and macrovascular complications as those with T1DM or T2DM [557]. Indeed, retinopathy, neuropathy, microangiopathy, and cardiovascular disease have been found to occur as frequently in T3cDM as in T1DM and T2DM [586,587,588].

##### Management and Treatment

Since T3cDM is due to endocrine and exocrine deficiencies, it requires individualized treatment. In patients with T3cDM, with mild hyperglycemia and dysfunction of β-cells, metformin may be considered based on studies in T2DM: since metformin is recommended as first-line oral therapy in T2DM, patients with T3cDM are initially treated with metformin. Since the principal defect in patients with T3cDM is insulin deficiency caused by impaired β-cell function, the preferred treatment in most cases is insulin therapy. However, the dosage is difficult to adjust because α-cell dysfunction negatively affects glucagon-mediated counter-regulation, resulting in a greater risk of hypoglycemia [561,585]. Unlike other forms of DM, because T3cDM is associated with pancreatic exocrine insufficiency (PEI), pancreatic enzyme replacement therapy (PERT) is essential. Patients with T3cDM also require specific nutrition counseling, including supplementation with fat-soluble vitamins (A, D, E, and K) [559,561]. It may also be worth trying oral insulin secretagogues in early T3cDM; however, therapy with thiazolidinediones may be associated with several side effects.

#### 2.2.6. Type 5 Diabetes Mellitus

Type 5 diabetes mellitus (T5DM), also known as *malnutrition-related diabetes* (MRD) or *malnutrition-related diabetes mellitus* (MRDM), is an under-recognized type of DM associated with chronic undernutrition [589]. It was initially identified in 1955 in Jamaica, and later in India, by Hugh-Jones; the author reports observing young, underweight people with IR who were not prone to ketoacidosis [590]. This form of DM was named J-type diabetes. In 1985, the new type of DM was officially recognized by the WHO as a distinct form of DM under the name *malnutrition-related diabetes* [591].

While T5DM was removed from its official classification in 1999 due to a lack of evidence [592], reports from many low-income and middle-income countries (LMICs), such as India, Pakistan, Bangladesh, Uganda, Ethiopia, Rwanda, and Indonesia, revealed the existence of an atypical phenotype of DM in lean individuals, which was associated with socioeconomic status and a history of prolonged undernutrition [593]. On 8 April 2025, at the 75th World Diabetes Congress, the International Diabetes Federation (IDE) reclassified it as a new type of DM, namely *malnutrition-related diabetes mellitus* or type 5 diabetes mellitus [594,595].

##### Prevalence of T5DM

T5DM is estimated to affect 20–30 million people worldwide, especially in regions of Asia and Africa [589,594,596], and its estimated prevalence ranges from 5% to 21.4% among patients with DM [597]. Some experts suggest that the true prevalence of T5DM might be higher, caused by the overestimation of the prevalence of T1DM in LMICs.

The prevalence of T5DM differs between regions and is closely related to local nutritional and socioeconomic conditions [598]. A substantial proportion of young and lean diabetic patients were described in South Asia, in countries such as Bangladesh, Sri Lanka, Pakistan, and India. These patients presented features of early-life undernutrition but were excluded from T1DM and T2DM [599]. Similar cases have been also detected in Jamaica, Nigeria, Uganda, Ethiopia, Rwanda, and South Korea [589]. Due to the lack of a unified nomenclature, however, several patients with MRDM have not been recognized [600].

##### Clinical Features and Diagnosis

Type five DM is diagnosed in individuals with a history of chronic undernutrition and is predominantly reported in LMICs, where food insecurity is higher compared to other countries. It is clinically significant in tropical regions, where malnutrition is prevalent [601]. In patients with T5DM, typically are observed following clinical features [589,602,603,604]. The patients are usually young, i.e., between 15 and 30 years of age, with most being male; T5DM is also detected in other age groups, although less frequently [605]. MRDR is reported in lean individuals (BMI < 19 kg/m^2^) who have a history of protein-energy malnutrition and micronutrient deficiency during the prenatal and postnatal periods and early childhood. The patients are often extremely lean and sometimes emaciated. Many present severe and persistent hyperglycemia with classical symptoms, such as polyuria and polydipsia. At diagnosis, blood glucose levels may be very high, but ketosis and DKD are absent.

Patients with T5DM require insulin (typically > 1.5 U/kg/day), although with poor response: glycemic control is therefore often difficult, despite high doses of insulin; these doses are needed due to reduced secretion, not IR. They demonstrate high fasting blood glucose levels (>200 mg/dL) without ketosis, even after insulin withdrawal. Many patients present with anemia, stunted growth, and vitamin and mineral deficits. These features are associated with nutritional deficiencies, such as protein-energy malnutrition and micronutrient insufficiencies. The symptoms of T5DM can be attributed to impaired β-cell function and exocrine damage caused by long-lasting malnutrition.

C-peptide levels are low but not absent, indicating primary β-cell dysfunction. Immunological markers, such as GADA or IA-2 antibodies, are typically absent; however, low-titer non-pathogenic antibodies may be observed [589,602,603,604]. Clinical identification of T5DM is complicated by the absence of validated and operational diagnostic criteria [593,606,607]. In many clinical settings, the absence of ketones in lean patients with DM is misleading. It is not uncommon for the patient to be diagnosed with T2DM based on non-ketotic presentation, the absence of autoantibodies testing, or low levels of C-peptide.

However, T5DM has certain important features, such as the absence of islet autoantibodies, a lower risk of DKD, and generally male sex, which may help to distinguish it from T1DM and T2DM [589]. The following points-based criteria may be used for a reliable diagnosis of T5DM: age of onset 10–30 years (1-point score), BMI < 19 kg/m^2^ (2 points), history of childhood malnutrition (1 point), childhood stigmata of past or present malnutrition of deficiency state (2 points), moderate to severe hyperglycemia, lack of proneness to ketosis in absence of stress (3 points), insulin required to achieved metabolic control but no dependence on insulin for prevention (2 points), and pancreatic calcification (3 points). An aggregated score greater 10 may suggest MRDR [589].

##### Pathophysiology of T5DM

The pathophysiology of T5DM is incompletely understood. It has been suggested that parental nutrition and metabolic status during critical periods, such as conception and early embryogenesis, influence the susceptibility of offspring to metabolic disorders [608]. Low intake of protein during pregnancy and lactation has a negative influence on fetal growth and disrupts the intracellular insulin signaling pathway and glucose metabolism, resulting in pancreatic β-cell dysfunction and an increased risk of DM. Impaired islet β-cell exocrine function is associated with impaired insulin secretion. Malnutrition can be also involved in the development of DM by impairing pancreatic development, which causes a reduced β-cell mass and impaired insulin secretion [589]. Another common feature of T5DM is exocrine pancreatic insufficiency (EPI), which impairs pancreatic development; this is associated with undernutrition during intrauterine life to early childhood, i.e., under five years of age [603].

In addition, chronic, persistent undernutrition can result in deficiencies in antioxidant compounds, such as vitamins A, C, and E, catalase, and glutathione peroxidase from fetal life into adulthood; this can impair the function of two transcription factors, PDX-1 and MafA, resulting in the defective expression of the insulin gene, impaired function of pancreatic β-cells, and reduced secretion of insulin [609]. PDX-1 may also be epigenetically altered by IUGR [600]. Protein or caloric restriction during pregnancy increases apoptosis and reduces proliferation in fetal pancreatic β-cells and hepatocytes, causing reduced β-cell mass, diminished production of insulin, and abnormal liver at birth [610]. Malnutrition can also disrupt the balance of pro-inflammatory cytokines and adipokines, such as IL-1β, TNF-α, and leptin, which play important roles in regulating insulin sensitivity, glucose metabolism, and β-cell function. Antioxidant deficiency and a low-protein diet result in increased apoptosis in islet cells and may cause elevated ROS and NO levels. The resulting oxidative stress also leads to mitochondrial dysfunction. A more comprehensive examination of the disturbances detected in patients with T5DM is given elsewhere [600,602,603,607].

##### Management and Therapy

Regular physical activity and balanced nutrition may improve insulin sensitivity and preserve pancreatic β-cell function. Patients are recommended to limit fat to under 30% of calories, reduce high-glycemic carbohydrates, consume high-protein–fiber foods and supplement with multivitamins, such as A, C, E, thiamine, B6, B12, and biotin, which are commonly deficient. Glycemic monitoring is also recommended because many patients require high doses of insulin.

#### 2.2.7. Cystic Fibrosis-Related Diabetes

Cystic fibrosis-related diabetes (CFRD) is the most common comorbidity in adults with cystic fibrosis (CF). It is an autosomal recessive disorder associated with mutations in the cystic fibrosis transmembrane conductance regulator gene (*CFTR*). The CFTR protein is an ion channel that transports chloride and bicarbonate across the cell membrane [611]. It is expressed in numerous tissues, particularly on the apical plasma membranes of epithelial cells, in the airways, small and large intestine, pancreatic and hepatobillary ducts, and the male reproductive tract [612]. Defective CFTR protein is associated with diseases affecting the respiratory, gastrointestinal, reproductive, sinonasal, and endocrine organs. This defect significantly increases the risk of morbidity and mortality [613]. One of the most common non-pulmonary complications is CFRD, which affects 20% of adolescents and up to 40–50% of adults aged over 30 years with CF [614]. Its prevalence doubles every decade by the third decade of life [611].

##### Prevalence of CFRD

Cystic fibrosis is estimated globally to affect between 70,000 and 100,000 people [615], in Caucasian populations with an incidence of 1 in 2500 births [616], and approximately 1 in 3000 live births in Europe [617].

The prevalence of CFRD varies between studies. While early disturbances in glucose metabolism become visible from a young age, it is uncommon under 10 years [618]. Abnormal glucose tolerance (AGT) can be observed in children under five years old [619]. CFRD itself is diagnosed in about 2% of children, and its prevalence increases with age, being identified in approximately 20% of adolescents and 30–50% of adult patients with CF [620,621]. This prevalence increases to 80% in patients aged over 40 years, who have severe genotypes [611]. Similarly, a European study performed in 2015 revealed a CFRD prevalence of 0.8% in patients under 10 years, 9.7% in patients aged 10–19 years, 24.1% in the 20–29 age group, and 32.7% in patients over 30 years of age [622].

##### Diagnosis of CFRD

The best method for screening and diagnosing CFRD is still believed to be challenging [612]. Currently, the screening test for CFRD recommended by the Cystic Fibrosis Foundation (CFF) and ADA is the OGTT, performed annually in all patients with CF aged 10 years and older [620,623]. Some studies suggest an earlier start, either from six years or after three years, following a diagnosis of CF. High-risk patients with a 1 h glucose level ≥ 200 mg/dL, should undergo more frequent screening for six months [624]. In 15% of patients with CF, hypoglycemia is detected during an OGTT (blood glucose levels <50 mg/dL). These patients may exhibit hypoglycemia caused by delayed initial insulin secretion, liver disease, malnutrition, gastrointestinal complications, and hormonal dysfunctions [625]. In adolescent patients with CF, a 1 h glucose value ≥ 140 mg/dL may also be an early indicator of CFRD risk [624].

The diagnosis of DM may also include FPG levels. This test was also recommended as one of the diagnostic criteria for CFRD by the ADA. However, FPG measurements cannot be relied on as the sole indicator for screening of CFRD [618]. The ADA also incorporated HbA1c as a potential method for screening and diagnosis of CFRD. Unfortunately, HbA1c is characterized by a sensitivity of only 50% and shows no correlation with mean blood glucose levels; as such, it is ill-suited for diagnosis of CFRD [618].

Other proposed screening methods include fractional serum fructosamine (FSF) and glycated albumin (GA). More detail is provided elsewhere [618].

##### Pathophysiology of CF and CFRD

Despite its high prevalence, the pathophysiology of CFRD remains incompletely understood. Its occurrence is multifactorial and heterogeneously associated with different factors, which have varying effects depending on the patient. It should be emphasized that CFRD is a unique form of DM: DKA is rare, the condition lacks any autoimmune processes, as is observed in T1DM, and the IR and macrovascular complications characteristic of T2DM are also rare. Also, classical symptoms observed in DM, such as polyuria and polydipsia, are often not observed in the early stage [626]. In contrast, CFRD patients may present some clinical features observed in other types of DM, such as insulin insufficiency, pancreatic inflammation, fatty infiltration, and impaired insulin secretion [619].

The development of glucose intolerance and CFRD in patients with CF appears to be insulin deficiency due to exocrine and endocrine pancreatic insufficiency caused by pancreatic islet β-cell dysfunction; this results in delayed or insufficient insulin secretion [611,627,628]. Evidence indicates that β-cell dysfunction begins in utero, while more substantial abnormalities are detected at five years old [629]. Pancreatic endocrine dysfunction begins in infants and is believed to correlate with reduced pancreatic β-cell mass and function; in particular, reduced conversion of proinsulin into insulin has been noted in young patients with CF [630]. Insulin deficiency is observed as post-prandial hyperglycemia due to delayed early insulin secretion [631]. After the loss of first-phase insulin secretion, it may be associated with IR, aging, and chronic systemic inflammation [628]. Interestingly, abnormal secretion of glucagon from pancreatic α-cells may be also involved in CF dysglycemia [632].

A major contributor to the development of CFRD is the loss of islet mass. A strong association has been noted between exocrine disease and the risk of developing CFRD. However, this risk is not solely accounted for by the presence of structural islet abnormalities, as indicated by the extent of pancreatic islet β-cell loss: postmortem investigations revealed ~ 50% loss of islet cell mass in patients with CFRD, as well as in patients with CF but without CFRD [611]. Also, autopsy studies showed poor correlation between β-cell mass and diagnosis of CFRD [633]. Furthermore, patients with CF can also develop CFRD, albeit at a much lower frequency, despite demonstrating pancreatic sufficiency and less pancreas damage than those with pancreatic insufficiency [633]. The development of CFRD has also been associated with β-cell dysfunction and insulin deficiency, obesity, inflammation of CF lung disease, epigenetic factors, and an impaired insulin signaling pathway [611,618,634]. The association between CF and CFRD is not completely explained and requires intensive research.

##### Complications

CFRD is associated with a fourfold increase in mortality due to chronic lung disease and gastrointestinal complications, including cirrhosis and malnutrition. Patients with CFRD are characterized by higher mortality compared to other patients with CF [635]. Patients with CF are at an elevated risk of lung disease: hyperglycemia exacerbates chronic inflammation, which influences lung infections and accelerates the decline of pulmonary function [636]. Patients with CFRD are more at risk of lung decline (first second of forced expiration in 1 s (FEV_1_ < 40%)) and for colonization by *Pseudomonas aeruginosa*. Early impairment in insulin secretion and glucose intolerance are both associated with a decline in lung function. As a result, patients are at a greater risk of hospitalization for lung complications and are more likely to require lung transplantation [626]. Patients with CFRD are more likely to demonstrate weight loss than CF patients without CFRD: this can be attributed to insulin deprivation causing protein catabolism and malnutrition and results in greater mortality [637].

Many CFRD patients demonstrate hypoglycemia. This is also noted during postprandial or fasting periods in CF patients without CFRD as an effect of impaired glucagon secretion [626]. In addition, CFRD patients have been found to exhibit microvascular complications such as albuminuria, nephropathy, retinopathy, and neuropathy, as well as microvascular complications resulting in vision defects, gastroparesis, and gastrointestinal symptoms [638], although macrovascular complications are rare. Patients with CFRD are at a higher risk of coronary arterial changes, arterial stiffness, and cardiovascular diseases [618,638].

##### Risk Factors for CFRD

The proposed risk factors for CFRD include age, genotype, and sex. The prevalence of CFRD rises from around 2% in children with CH to around 19% in adolescents and up to 50% in adult patients [636]. Data from genome-wide association studies (GWAS) indicate only a weak genetic association between CFRD and T1DM, while a family history of T2DM increases the risk for CFRD by threefold [639,640]. The risk of CFRD closely correlates with certain genes associated with T2DM: *TCF7L2*, *SLC26A9*, *CDKAL1*, *CDKN2A/B*, and *IGF2BP2* [634]. It has been suggested that variants of *SLC26A9* are specific to CFRD, while other variants are associated with both CFRD and T2DM [639]. Interestingly, this type of DM is also more often diagnosed in women than men [641]; this may be associated with the fact that bacterial infections occur earlier and more frequently in women than men, and these often cause systemic inflammation. Systemic infection and corticosteroid therapy are known to contribute to IR and CFRD [611,618,619]. This is discussed in more detail elsewhere [619,634].

##### Management and Therapy of CFRD

Again, CFRD responds best to a personalized approach based on the clinical presentation and severity of each case [618]. The treatment strategy for CFRD comprises several components, such as pharmacological therapy, nutritional support, and physical activity.

The primary therapy recommended for adolescents with CFRD, and the only pharmacotherapy for children, is insulin [628]; however, other oral glucose-lowering drugs have been proposed. One alternative is repaglinide [628]. This therapy is recommended for patients without weight loss, pulmonary decline, or persistent hyperglycemia; however, repaglinide may increase the risk of hepatic toxicity. In addition, DPP-4, metformin, and GLP-1 receptor agonists have also been used to lower blood glucose levels. Nutritional treatment and physical activity have been proposed as non-pharmacological therapies [618,634,638].

Alternatively, CFRD modulators have been proposed as a treatment. These medications target defects in CFTR protein and have been found to be effective in improving lung function, reducing gastrointestinal issues, and improving weight [626]. The first generation of modulators includes ivacaftor, which improves the channel function of CFTR. The second generation includes combination therapy based on ivacaftor plus corrector lumacaftor (LUM-IVA) or ivacaftor plus corrector tezacaftor (TEZ-IVA). These modulators are used in patients in which CF is due to mutation F508del. The third generation is ET1 therapy, combining tezacaftor with ivacaftor and additional corrector elexacaftor. ET1 improves lung function [618,626,634,638].

The precise mechanisms by which CFTR modulator therapy influence the pathogenesis of CFRD remain incompletely understood [618].

### 2.3. Conceptual and Metaphorical Types of Diabetes Mellitus

Several other types of diabetes mellitus exist, which are not officially classified.

#### 2.3.1. Type 3 Diabetes Mellitus

The term *type 3 diabetes mellitus* (T3DM) suggests a pathological association between T2DM and Alzheimer’s disease (AD), insofar that IR in the brain plays a role in the metabolic damage associated with neuron degeneration. In some studies, the association between AD and T2DM is regarded as so strong that the term *diabetes of the brain* is used to describe it. It should be noted though that although *type 3 diabetes mellitus* has been suggested to describe the association between DM and AD, it remains a working hypothesis rather than an accepted diagnosis. Indeed, while IR is believed to be involved in neurodegeneration, other studies indicate that IR can arise secondarily from amyloid and tau pathologies [642]. Therefore, it remains unclear whether IR a primary driver or secondary symptom of neurodegeneration [643].

AD is an age-related, progressive neurodegenerative disease causing dementia. The prevalence of AD doubles every five years among individuals aged over 65 years. The risk of AD increases with age [644]. The WHO and the World Alzheimer Report (WAR) estimate that over 55 million individuals have been diagnosed with dementia, and more than 10 million new cases are identified each year. It is suggested that dementia will be diagnosed in 78 million patients in 2030 and in 139 million in 2050 [645,646,647]. Studies indicate that AD is the fifth most common cause of death among individuals aged over 65 years [648].

Diabetes, particularly T2DM, is extremely common among the elderly. The global prevalence of DM in 2019 was estimated to be 9.3% (463 million individuals), rising to 10.2% (578 million) by 2020; this value is expected to increase by 10.9% (700 million) by 2045 [649] and another 12.5% (853 million) by 2050 [596]. Studies suggest that insulin resistance may be associated with clinical symptoms of AD, such as memory loss and cognitive deterioration [650]. Research also indicates that T2DM is a common risk factor for AD [651]. A meta-analysis of prospective observational studies found patients with DM to have over a 70% greater risk of all types of dementia and almost a 60% greater risk of developing AD compared to non-diabetic individuals [652]. Another meta-analysis of prospective studies revealed a 1.25- to 1.91-fold increased risk of cognitive impairment and dementia [653] and a 59% increased risk of dementia in patients with DM compared to a healthy control group [654].

##### Role of Insulin in the Brain

Insulin is an anabolic hormone synthesized in the β-cells of the islets of Langerhans, whose main role is the regulation of blood glucose levels. The presence of insulin in the brain was first described in 1978 [655]. Insulin mRNA has also been found to be expressed in the brain and in cultured neurons but not in glial cells [656,657]. This observation may suggest that the brain can produce insulin [658,659], although the low amount of insulin mRNA is insufficient to confirm production [660,661]. The amount of insulin in the brain can be increased by drawing on the insulin circulating in the blood: it can cross the blood–brain barrier by a selective, receptor-mediated active transport system [662]. This transport rate can be changed by several factors, including AD [663].

The brain appears to be an insulin-sensitive organ. Insulin plays an important role in neurogenesis by regulating the proliferation, differentiation, and survival of neuronal stem cells [664]. The insulin signaling pathway plays various roles in maintaining circuit regulation, synaptic plasticity, certain cognitive functions, memory consolidation, and control of neuronal and glial metabolism [665,666]. Abundant INSR has been detected in the brain regions associated with memory, cognitive function, and metabolic regulation, such as the hippocampus, cerebral cortex, hypothalamus, and olfactory bulb [667,668]. Insulin acts as a facilitator of synaptic plasticity, an important process in memory formation and learning [669,670]. It also improves the efficiency of neural network functioning by enhancing communication between neurons. Insulin regulates the levels of neurotransmitters influencing brain activity, mood, and behavior. It has also demonstrated neuroprotective properties by protecting neurons from damage, enhancing neuron survival, and reducing oxidative stress and inflammation. In addition, by facilitating glucose uptake, insulin plays a role in brain energy metabolism [668,671,672].

##### Intracellular Insulin Signaling Pathway in Healthy Brains

In the brain, the insulin signaling pathway regulates a number of cellular mechanisms and metabolic elements. Its effect begins by binding to the INSR, initiating a cascade of downstream signaling pathways [673]. As a result, this binding induces various conformational changes, resulting in the autophosphorylation of several tyrosine residues in the INSR β-subunit. The activated INSR molecule induces the phosphorylation of adapter proteins, such as IRS. In humans, there are six IRS proteins (IRS-1 to IRS-6); of these, IRS-1 and IRS-2 play an important role in insulin signaling pathways, and both are also closely associated with the development of IR [663]. The activated IRS proteins activate key signaling pathways, such as phosphoinositide 3 kinase/protein kinase B (PI3K/AKT), the mitogen-activated protein kinase (MAPK)/extracellular signal-regulated kinases (ERK), and the mammalian target of rapamycin (mTOR) pathway [674].

The PI3K/AKT pathway is associated with the regulation of GLUT3 and phosphofructokinase-1 expression. GLUT3, largely expressed in neurons, regulates glucose and energy levels. Its activation causes the translocation of GLUT4 from the intracellular compartment to the plasma membrane of neurons in the hippocampus, enhancing cognitive performance [675]. The intracellular MAPK/ERK pathway is associated with memory and neuroprotection and helps regulate glucose and lipid metabolism in the mediobasal hypothalamus and the dorsal vagal complex [676]. The MAPK/ERK pathway may be involved in neuroinflammation, resulting in the accumulation of amyloid plaques, which perpetuate symptoms of AD [677]. Finally, the AMPK signaling pathway is a regulator of metabolic homeostasis. Its activation inhibits mTOR, preventing excessive cellular growth and promoting autophagy. The pathway also decreases hyperglycemia by suppressing gluconeogenesis. Stimulating the translocation of GLUT4 to the plasma membrane enhances glucose uptake. The AMPK pathway suppresses the inflammation involved in the pathophysiology of T2DM and AD [678]. In the hypothalamus, AMPK activity is inhibited by insulin and glucose [679]. The AMPK pathway also plays a significant role in neuroprotection [679]. Finally, the mTOR pathway, activated by PI3K/AKT signaling, negatively regulates autophagy. It is believed that the inhibition of the mTOR pathway may result in neuroprotective effects in AD by stimulating the removal of damaged cells [680]; indeed, a number of neurodegenerative diseases, such as AD, are characterized by a lack of autophagy [40,673,681,682].

##### Pathophysiology of AD

The first description of AD was made in 1906 by the German psychiatrist and neuropathologist Alois Alzheimer, when he identified *miliary foci* in female patients [683]. Alzheimer’s disease is a neurodegenerative disease that occurs in the elderly population. Its etiology is multifactorial, including genetic predisposition, advanced age, and neuroinflammation; however, various other factors, such as gut microbiota composition, may also play a role in its development [645]. The condition is brought on by the early onset of aberrant Tau hyperphosphorylation and formation of amyloid-β (Aβ) plaques; these cause neuronal dysfunction, resulting in a progressive loss of hippocampal neurons and impaired synaptic transmission as an effect of neurotransmitter dysregulation [684].

- Forms of AD

Although AD is primarily diagnosed in patients over the age of 65, it has been classified into different forms depending on the age of onset, *viz*. early-onset AD (EOAD), also called young-onset AD (YOAD), and late-onset AD (LOAD).

Approximately 5–6% of AD cases are classed as EOAD, generally detected in individuals aged 45–65 years [685]. This form may result from head trauma, cardiovascular conditions, chronic schizophrenia, genetic predispositions, or Down syndrome. It may be also associated with lifestyle factors, such as poor diet, smoking, or alcohol use [686]. Regarding its genetic inheritance pattern, EOAD can be categorized as mendelian early-onset AD (mEOAD) and non-mendelian early-onset AD (nmEOAD). mEOAD is relatively rare, and it is caused by specific mutations in genes such as *APP*, which codes the amyloid precursor protein (APP), and *PSEN1* and *PSEN*2, which code presenilin 1 (PSEN1) and presenilin 2 (PSEN2). This form of EOAD is usually diagnosed in patients under 65 years old. nmEOAD does not have a clear genetic inheritance pattern and is influenced by environmental and genetic factors. It is more common than mEOAD and exhibits a greater variability in age of onset and disease progression [687].

In contrast, LOAD is diagnosed in around 94–95% of AD patients aged over 65 years old [688]. Its risk factors include vascular disease, sleep disturbances, such as insomnia and reduced deep sleep, genetic factors, especially the apolipoprotein E epsilon 4 (*APOE-ε4*) allele, and changes associated with brain aging [688,689,690].

Clinically, there are two types of AD [691]: *late onset* or *sporadic AD* (sAD) and *early-onset* or *familial AD* (fAD) [673,681,692]: sAD is diagnosed in 90–95% of patients with AD aged ≥ 65 years. The genetics of sAD are more complex [693]. A significant risk factor for the development of sAD is the *APOE-ε4* gene. The presence of two copies of the gene increases the risk of AD twelvefold, while one copy increases it fourfold [694]. It is suggested that the gene is carried by 50–60% of individuals and that other factors are also involved in the risk of developing AD [695].

In 5–10% of patients, AD occurs in response to rare genetic mutations. These patients are diagnosed with fAD. This type of AD is diagnosed in younger patients, i.e., those in their thirties, forties, or fifties [692]. This type is associated with mutations in three genes: *APP, PSEN1*, and *PSEN2*. While other mutations may also have an influence, they remain unknown [691].

- Brain insulin resistance and AD

Insulin resistance is associated with decreased sensitivity of the insulin receptor, resulting in decreased PI3K activation and impaired AKT phosphorylation [696]. This results in the dephosphorylation and activation of glycogen synthase kinase-3 beta (GSK-3β), which promotes Tau phosphorylation and the production of neurofibrillary tangles (NFTs) [697]. Dysfunction of AKT can prevent the inhibition of forkhead box O1 (FOXO1), reducing cell survival and increasing apoptosis [40,673,681,682,698].

- Insulin resistance and Tau protein

Tau protein is a soluble microtubule-associated protein responsible for microtubule stability and regulating intracellular signaling pathway [699,700]. In humans, the Tau protein is encoded by the microtubule-associated protein Tau (*MAPT*) gene, located on chromosome 17q21-q22. Tau protein is expressed in various regions throughout the body, not just in the brain [701,702]. In the peripheral nervous system, Tau protein is involved in several important processes [703,704,705], such as the regulation of glucose-stimulated insulin secretion [705]. The Tau protein hypothesis proposes that normal Tau protein is transformed into paired helical fibers (PHF) and NFTs by Tau protein hyperphosphorylation, resulting in the development of AD [706]. The misfolded Tau proteins aggregated into NFTs are not broken down by autophagy. The aggregates disrupt neuronal function by sequestering normal Tau protein and other microtubule-associated proteins [707]. Tau protein can induce IR and T2DM [704]. The phosphorylation of the Tau protein is associated with Tau protein aggregation; however, in physiological states, Tau hyperphosphorylation and misfolding is prevented by the protein phosphatase 2A (PP2A).

However, Tau hyperphosphorylation is strongly influenced by impaired glucose metabolism and insulin dysfunction. These abnormalities also affect the MAPK and AKT signaling pathways, which play an important role in Tau regulation [645,708]. Tau phosphorylation is also regulated by GSK-3β, whose activity is, in turn, regulated by the insulin signaling pathway [709,710]; GSK-3β overactivity can cause hyperphosphorylation, which reduces Tau protein expression by impairing the insulin signaling pathway. In contrast, GSK-3β activity is suppressed by insulin [711]. Tau protein in the pancreas was found to be hyperphosphorylated in animal models of AD and T2DM [712], and hyperphosphorylated Tau protein has been detected in the pancreas of patients with T2DM [651]. Hyperglycemia, a characteristic feature of DM, can induce hyperphosphorylation of Tau protein and NFT accumulation [713].

- Insulin resistance and β-amyloidosis

The pathogenesis of AD is initiated by impaired synapse function caused by the accumulation of amyloid β (Aβ), produced from APP. Its gene is located on chromosome 21 [714,715]. As patients with Down syndrome demonstrate trisomy 21 (47, XX +21 or 47, XY +21), they have an increased risk of developing AD due to the additional copy of the *APP* gene, resulting in its overexpression [716]. Aβ is a soluble protein involved in synaptic plasticity, memory processes associated with the hippocampus, neuroprotection, cellular metabolism, and survival [717]. APP is a widely expressed transmembrane protein, which is most abundant in the CNS and in synapses. It plays a role as a cell surface protein, and it is involved in the regulation of synapse formation, neural plasticity, antimicrobial activity, and iron export. In association with amyloid precursor-like protein 2 (APLP), APP is involved in maintaining learning and memory [718].

Aβ is synthesized in the endoplasmic reticulum and then transported to Golgi apparatus; after maturation, it is transported to the plasma membrane. Following this, APP synthesis can take place via two pathways: 10% is produced by the amyloidogenic (plaque forming) pathway and the remaining 90% by the non-amyloidogenic (non-plaque forming or secretory) pathway [719,720]. Insulin promotes the non-amyloidogenic pathway by regulating phosphorylation. When the insulin signaling pathway is impaired, the amyloidogenic pathway is stimulated, resulting in increased accumulation of pathological Aβ [721].

In the non-amyloidogenic pathway, APP is cleaved by α-secretase, resulting in the production of a C-terminal fragment α (CTFα, C83) and a soluble N-terminal fragment α (sAPPα). CTFα is then cleaved by γ-secretase, resulting in a smaller C-terminal fragment (C3) [722,723]. This process is strongly dependent on α-secretase, which prevents the generation of Aβ peptide.

The amyloidogenic pathway occurs within the endosome in an acidic microenvironment. In the pathway, β-secretase cleaves APP into a smaller N-terminal (sAPPβ) fragment and a longer C-terminal fragment, which contains the full amyloidogenic amino acid sequence (CFTβ, C99). CFTβ is then cleaved by γ-secretase, resulting in the formation of an APP intracellular cytoplasmic domain (AICD) and Aβ fragments. These fragments are released into the extracellular environment, where the Aβ fragments rapidly form oligomers, prefibrillary aggregates, and fibrils, ultimately forming β-amyloid plaques [722,723].

The Aβ peptide chain consists of 38 (Aβ_38_), 40 (Aβ_40_), and 42 (Aβ_42_) amino acids. Increased expression of the *APP* gene, combined with altered proteolysis, causes the accumulation of 40 or 42 aa length Aβ polypeptides. In the case of fAD, mutations in the *APP, PSEN1*, and *PSEN2* genes and the inheritance of the *APOE-ε4* allele alter the role of γ-secretase, increasing the production of Aβ_42_ peptides [724]. These mutations promote the synthesis and deposition of Aβ peptides in the brain. Native APP plays a positive role in the process of memory and learning by supporting synapse activity and dendritic spine formation [725,726]. In a healthy brain, neuronal activity causes the release of Aβ to the extracellular space, and its levels are regulated by local proteases [727]. Amyloid plaques block the signaling pathway and cell connections, resulting in cell death.

In animal studies, intracerebral administration of neurotoxic Aβ was found to cause memory and learning deficits, similar to those observed in AD patients. This suggests that impairments in memory and learning are associated with disrupted synaptic plasticity due to intraneuronal accumulation of Aβ [728]. Also, hippocampal administration of Aβ oligomers in rodents causes synaptic loss and neuronal dysfunction, resulting in memory impairment [729]. It is also suggested that negative effects of Aβ on neurons are caused by the activation of the immune–inflammatory reaction in glial cells, resulting in the phagocytosis of neuronal and synaptic structures [730].

The metabolism of APP is regulated by insulin, which controls the balance between the anabolism and catabolism of Aβ. Low levels of insulin, or its lack of activity, may result in increased NFT formation, oxidative cell damage, and elevated Aβ and amyloid plaque levels in the brain [731].

Insulin plays an important role in the clearance of Aβ and prevents the formation of fibrils and plaques and their extracellular accumulation. Low levels of insulin are associated with increased NFT formation, elevated Aβ levels, and the formation of amyloid plaques. In contrast, high insulin levels cause prolonged hyperinsulinemia. One factor influencing insulin level is the insulin-degrading enzyme (IDE); IDE is a thiol zinc-metallo-endopeptidase that regulates the levels of insulin and Aβ in the brain by cleaving insulin, Aβ, glucagon, and calcitonin.

Insulin also prevents the formation of Aβ fibrils, stimulates the internalization of Aβ oligomers, and blocks their binding to neurons, protecting synapses against Aβ oligomers [732]. Unfortunately, the protective effects of insulin have been described mainly in experiments and not in clinical studies. These protective effects against Aβ accumulation are impaired by IR, resulting in the downregulation of insulin expression: Aβ peptides inhibit the binding of insulin to its receptor and decrease INSR autophosphorylation, which interferes with the insulin signaling pathway [733].

It has been suggested that IDE plays a role in the link between IR and AD [734]. In conditions of IR, IDE is involved in the degradation of excess insulin, reducing its availability for Aβ degradation [651,735,736]; this results in the accumulation of Aβ and formation of extracellular plaques [673]. Indeed, high doses of insulin in rats were found to significantly reduce Aβ clearance [737]. Other animal studies indicate that IR increases the activity of γ-secretase, increasing the levels of Aβ in the brain and decreasing the activity of IDE [700].

An association has also been noted between the *APOE-ε4* gene and IDE expression. Carriers with AD demonstrate reduced expression of IDE in the hippocampus and some other areas of the brain [738]. Additional observations confirm reduced IDE activity in patients with genetically inherited forms AD [739,740].

- Advanced Glycation End Products and AD

Advanced glycation end products (AGEs) are also believed to contribute to the development of AD. These are peptides or protein molecules that undergo non-enzymatic glycosylation (glycation) following exposure to sugars as a result of the Maillard reaction [741,742]. While AGEs arise as byproducts of normal aging, the rate of generation is increased in patients with T1DM and T2DM [697,743,744] due to hyperglycemia.

AGEs accumulate in various cells. In the CNS, they interact with their receptors (RAGEs) expressed in neuronal cells, microglia, astrocytes, and brain endothelial cells [745]. Higher levels are detected in patients with AD and T2DM [673]. AGEs are also expressed in neuron-derived exosomes, which are involved in the development of AD [746]. These molecules stimulate the aggregation of amyloid oligomers, which is thought to be responsible for the neurotoxic consequences observed in AD patients [747]. Glycation enhances Aβ aggregation. AGEs are also involved in Tau protein hyperphosphorylation and PHF formation [748].

It was also suggested that AGEs may coexist with the *APOE-ε4* gene in the amyloid plaques of patients with dementia, particularly those who suffering from AD [748]. AGEs generate free radicals by triggering the activity of NADPH oxidase (NOX), the major source of ROS in glial cells and neurons [749]. Furthermore, AGEs demonstrate neurotoxic properties and have been found to decrease cell viability in cortical neurons [750].

The interaction of RAGEs and ligands may increase the production of beta secretase-1, also known as beta-site APP-cleaving enzyme (BACE1), promoting the generation of Aβ from APP by the amyloidogenic route [751]. RAGEs, in response to Aβ, re involved in the migration of monocytes in human brain endothelial cells in both healthy subjects and patients with AD [752]. After binding to Aβ, RAGEs can transport brain-derived Aβ across the BBB in the peripheral circulation [753] and back again [754], thus supporting the neurodegenerative pathway.

Binding to the Aβ peptide upregulates the nuclear factor-kappa beta (NFκβ) transcription factor, which plays a role as a mediator in inflammation [755]. This results in the development of diabetic vascular injury and stimulates the release of cytokines, such as TNF-α and IL-6, from microglia. This, in turn, causes neuronal damage due to the expression of macrophage-colony stimulating factor (M-CSF) [754,756]. Hence, it is suggested that AGEs serve as links between IR and neurodegeneration, especially in a hyperglycemic state [749].

- Neuroinflammation and AD

According to de la Monte, neuroinflammation is defined as “the presence of activated microglia and astrocytes which cause injury through expression and release of pro-inflammatory cytokines, chemokines, and complement increased generation of membrane fatty acids, eicosanoids, lipid peroxidation products, and reactive oxygen and reactive nitrogen species (RNS)” [642]. Acute inflammation can initially play a protective role, while chronic inflammation in the brain contributes to the progression of AD [645].

Innate immunity in the brain is associated with astrocytes and microglia. The astrocytes of patients with AD demonstrate morphological and functional differences to those in patients without AD. For example, the astrocytes or AD patients are more reactive, resulting in a greater release of pro-inflammatory cytokines such as IL-1β and TNF-α [711,757,758]. These functionally impaired astrocytes are less effective in clearing excess glutamate from the synaptic cleft, causing excitotoxicity and neuronal damage [645]. In addition, the activation of microglial cells, which represent the immune system of the mammalian brain [727], has also been suggested as a main cause of clinical inflammation and, potentially, brain pathology [759].

Neuroinflammation is a characteristic feature in several neurodegenerative diseases, including AD. In AD, it is believed to exacerbate neurodegeneration [757]. It has also been found to play an important role in neuronal injury and cholinergic dysfunction [760], as well as oxidative stress [761]; it also increases the production of ROS and RNS, which damage nerve terminals, resulting in synapse dysfunction [762]. Neuroinflammation is also associated with Tau protein hyperphosphorylation [763] and β-amyloid accumulation [764]. While IR can be exacerbated by chronic inflammation, neuroinflammation is an integral feature of AD pathophysiology [662,765,766]; even so, its role in the development of IR in the brain, and in T2DM and AD, has not been fully established [700]. Currently, no evidence indicates that anti-inflammatory therapies work against AD [700].

- Is AD T3DM?

There is growing evidence that AD may be linked to DM, in particular T2DM [666,710,767,768,769,770], as well as T1DM [769,770]. In addition, while T3DM is not officially recognized as a formal diagnosis in the medical field, its presence may suggest a potential association between DM and AD.

One of the main symptoms of T2DM is IR. In the brain, IR also plays an important role in AD by disrupting neuronal survival, synaptic plasticity, and memory regulation, particularly in the hippocampus and cortex [771]. It has also been found to disrupt the intracellular insulin signaling pathway, thus reducing AKT activity. This, in turn, releases GSK-3β inhibition, causing excessive Tau phosphorylation and neurofibrillary tangle formation [705], the main cause of AD. However, it remains unresolved whether IR exists as a primary driver of AD or as a secondary effect [663,771].

Impaired insulin production and sensitivity, due to IR, can be associated with clinical signals of AD, such as memory loss and cognitive deterioration [650]. In addition, T2DM is a common risk factor for AD [651], and some of its clinical symptoms, in patients with DM, are problems with memory processing, brain morphology, and synaptic communication, owing to IR in the brain and regional brain glucose metabolism [772]. Notably, the therapeutic drugs used in T2DM, such as metformin, GLP-1 receptor agonists, and intranasal insulin, also have neuroprotective effects in patients with AD [711]. There are also other results regarding GLP-1 receptor agonists. The EVOKE and EVOKE+ trials investigated the efficacy and safety of oral semaglutide, a GLP-1 receptor agonist, in patients with early-stage symptomatic AD. The obtained results revealed that the use of semaglutide was not efficacious in slowing clinical progression in these patients [773]. It is important to note that these findings do not rule out all potential applications of this drug class. Several other similarities between DM and AD are noted in other studies [643,645,663,697,710].

Indeed, de la Monte and Wands note “the term *type 3 diabetes* accurately reflects the fact that AD represents a form of diabetes that selectively involves the brain and has molecular and biochemical features that overlap with both type 1 diabetes mellitus and type 2 diabetes mellitus” [774].

#### 2.3.2. Type 4 Diabetes Mellitus

Type 4 diabetes mellitus (T4DM) was first diagnosed in 2015. It represents the first confirmation of the relationship between aging and DM. In 2017, the new disorder was named Type 4 DM; however, it remains under study until the relationship between age-related changes and insulin function disorders is better understood, so it is still not officially classified. Because T4DM is still an unofficial classification of DM, there is not much information surrounding it. Unlike T1DM and T2DM, which stem from an overactive immune system and excess weight, respectively, T4DM is consequence of aging. It is diagnosed in elderly individuals with IR, who are typically lean, with moderate weight [775]. It primarily affects older adults, particularly those >65 years of age, and rarely younger people. T4DM has a different set of risk factors to other types of DM. It has been suggested that “metabolic syndrome and IR were associated [*with an*] increased risk of frailty” [776]. Indeed, T4DM has been found to affect up to 20% of newly diagnosed patients with DM aged over 65 years [777]; however, T4DM often remains undiagnosed, as patients lack the classical risk factors, such as obesity.

##### Clinical Symptoms and Diagnosis

The clinical symptoms of T4DM do not differ from those of other types of DM. Common symptoms include fatigue, increased thirst, increased hunger, blurry vision, frequent urination, delayed healing of sores, unintentional weight loss, numbness and tingling in extremities, and dry mouth. As these symptoms are generally detected in individuals with moderate weight, clinicians may not suspect diabetes. These symptoms may be also associated with aging.

As there is no specific test for T4DM, there is no official diagnosis. Diagnosing T4DM typically includes non-specific tests used for other types of DM such as HbA1c, fasting blood glucose test, oral tolerance test, and random blood sugar tests [778,779]. The described glycemic tests can establish a diagnosis of DM but cannot confirm a distinct “Type 4” category.

##### Pathophysiology of T4DM

The development of T4DM is usually based around the activity of regulatory T cells (Tregs), which control inflammation and are responsible for the aging processes. Studies on animal models, and then confirmed in humans, indicate that excess production and availability of Treg cells in the body can cause the onset of T4DM. Patients with T4DM exhibit high levels of regulatory T lymphocytes within adipose tissue. As people age, these cells can accumulate in fat, leading to increased inflammation. The accumulation of regulatory T lymphocytes within fat causes the fat to accumulate within viscera, leading to IR [777]. In patients with T4DM, the development of IR in the brain may cause glaucoma [780].

T4DM can cause various complications, such as cardiovascular disease, kidney problems, issues related to vision, including retinopathy, foot problems, and diabetic ulcers.

##### Management and Therapy

Unfortunately, there is no specific treatment for T4DM. Treatment generally proceeds in a similar way to T2DM, using drugs, such as metformin, sulfonylureas, DPP-4 inhibitors, SGLT2 inhibitors, and GLP-1 receptor agonists. Insulin may also be used to achieve normal blood glucose levels if necessary [778,779]. Further research is needed on both the nature and treatment of T4DM.

## 3. Conclusions and Future Perspectives

Diabetes mellitus comprises a group of non-communicable metabolic disorders that affect multiple organs and tissues and represents one of the most prevalent diseases worldwide. These diseases include four main types: cardiovascular diseases, cancer, chronic respiratory diseases, and diabetes mellitus. The prevalence of DM is believed to be rapidly increasing and is estimated to affect over 500 million adults. This growth has been attributed to various risk factors, including a sedentary lifestyle with a lack of physical activity, highly caloric diet, overweight and obesity, alcohol, and smoking cigarettes.

Research has divided DM into a number of common types, such as T1DM, T2DM, and GDM, as well as various rarer types, such as LADA, MODY, T3cDM, and CFRD. Some other conceptual or metaphoric types such as T3DM and T4M have also been proposed, which have not yet been awarded a formal diagnosis. Nevertheless, each type is characterized by specific clinical symptoms, causes, pathophysiology, management, and therapy, although in some types, the precise pathophysiological mechanisms remain unclear. Therefore, there continues to be a need for further research into the nature of their mechanisms, the associations between their cause and symptoms, and the pathways influencing their occurrence; their findings will play an invaluable part in lifting the burden of DM and its complications. Such research demands close cooperation between doctors and scientists, an intensive search for new therapies, and the understanding the values of a healthy lifestyle.

## Figures and Tables

**Table 1:** Classification of diabetes mellitus in dependence on incidence [3,6,7,8].

Incidence of DM	Type of DM
Common types of diabetes mellitus	Type 2 diabetes mellitus (T2DM), also known as non-insulin-dependent diabetes mellitus (NIDDM) or adult-onset diabetes mellitus, is the most common type of DM. It accounts for about 90% of all cases of DM.
	Type 1 diabetes mellitus (T1DM) was previously called *insulin-dependent diabetes* or *juvenile-onset diabetes*. It accounts about 5–10% of all cases of DM.
	Gestational diabetes mellitus (GDM) is defined as glucose intolerance of various degrees that is first detected during pregnancy. The prevalence of GDM depends on population type, ethnicity, age, body composition, and diagnostic criteria. Worldwide, its prevalence ranges from 5% to 25%.
Rare types of diabetes mellitus	Latent autoimmune diabetes in adults (LADA) is referred to as diabetes mellitus type 1.5 (T1.5DM), slow-onset autoimmune diabetes (SAID), slowly progressive insulin-dependent diabetes (SIDDM), double diabetes, late type 1 diabetes, youth-onset diabetes of maturity, latent-onset type 1 diabetes, and antibody-positive non-insulin-dependent diabetes. It accounts for approximately 2–12% of diabetic patients.
	Maturity-onset diabetes of the young (MODY), also known as *monogenic diabetes*, accounts for 1–5% of all cases of DM in Europeans. There are 14 subtypes of MODY.
	Neonatal diabetes mellitus (NDM), also known as *congenital diabetes* or *diabetes of infancy*, the second type of monogenic DM, has been attributed to a monogenic defect. It is estimated to account for 1–5% of all DM cases. NDM is indicated by insulin-requiring hyperglycemia or where the onset of diabetes occurs before the age of 6 months.
	Steroid-induced diabetes mellitus (SIDM) is defined as “abnormally elevated blood glucose associated with the use of glucocorticoids in patients with or without a previous history of DM”. However, SIDM is common in the adult population, since as many as 1–8% of all adult patients are receiving glucocorticoids; in children, it is reported as rare; however, it has been suggested that “SIDM may not be so rare in the pediatric population as once thought”.
	Type 3c diabetes mellitus (T3cDM), also known as *pancreatogenic DM*, occurs secondary to pancreatic diseases, which affect the pancreas exocrine compartment and/or composite (exocrine and endocrine) pancreas. T3cDM accounts for 5–10% of DM cases worldwide. Some data suggest that pancreatogenic diabetes accounts for 1–9% of all cases of DM, whereas others indicate it to be present in approximately 2% of cases.
	Type 5 diabetes mellitus (T5DM), also known as *malnutrition-related diabetes* (MRD) or *malnutrition-related diabetes mellitus* (MRDM), is an under-recognized type of DM associated with chronic undernutrition. The International Diabetes Federation (IDE) reclassified it as a new type of DM, namely *malnutrition-related diabetes mellitus* or type 5 diabetes mellitus. Its estimated prevalence ranges from 5% to 21.4% among patients with DM.
	Cystic fibrosis-related diabetes (CFRD) is the most common comorbidity in adults with cystic fibrosis (CF). It is diagnosed in 2% of children with CF, approximately 20% of adolescents, and 30–50% of adults with CF.
Conceptual/metaphoric, not formal diagnosis types	Type 3 diabetes mellitus (T3DM), together with *diabetes of the brain*, has been proposed as a term to represent the pathological association between T2DM and Alzheimer’s disease (AD). However, its use remains a working hypothesis rather than accepted diagnosis. It currently remains unclear whether insulin resistance may play a role in neurodegeneration or whether insulin resistance can arise secondarily from amyloid and tau pathologies.
	Type 4 diabetes mellitus (T4DM), first proposed as an entity in 2017, is associated with aging; however, the classification remains unofficial while data are gathered on the relationship between age-related changes and insulin function disorders. T4DM may affect up to 20% of newly diagnosed cases of DM.

**Table 2:** Clinical differences between LADA, T1DM, and T2DM [307,308].

Features	LADA	T1DM	T2DM
Age of diagnosis	Usually between 30 and 50 years	Usually in childhood and very rare in adulthood	Adulthood
Insulin resistance	Usually high	Absent	Usually high
Function of β-cells	Decreased	Rapid β-cell destruction and loss of function	Normal or slightly increased
Insulin therapy	Usually after six months after diagnosis	Required at the time of diagnosis	Absent or might be delayed months to years after diagnosis
BMI	Normal, rarely obese	Normal or underweight	Overweight or obese
Risk of complications	Increased risk of cardiovascular diseases	Increased risk for cardiovascular diseases	Increased risk for cardiovascular diseases
Family history	Not necessary; patients have a higher prevalence of personal or family history of autoimmune disease	Not necessary	Frequently present
HLA susceptibility	Higher	Increased	Slightly increased
Autoimmunity	Slightly raised	Slightly raised	Absent

## Data Availability

No new data were created or analyzed in this study. Data sharing is not applicable to this article.
